# Digital Health Applications for Mental Disorders in Germany Among Users and Nonusers on Actual and Intended Use, Self-Reported Completion, and Reasons for Discontinuation: Cross-Sectional Survey

**DOI:** 10.2196/99464

**Published:** 2026-10-29

**Authors:** Klemens Höfer, Jürgen Wasem, Anna Bußmann, Carsten Volland, Pascal Raszke, Sarah Schlierenkamp, Luisa Friedrich, Josepha Katzmann, Udo Schneider, Anja Wadeck, Sophia Zander, Carina Abels

**Affiliations:** 1 Institute for Health Care Management and Research University of Duisburg-Essen Essen, North Rhine-Westphalia Germany; 2 Essener Forschungsinstitut für Medizinmanagement GmbH Essen, North Rhine-Westphalia Germany; 3 Deutsche PsychotherapeutenVereinigung Berlin Germany; 4 Techniker Krankenkasse (Germany) Hamburg, Hamburg Germany

**Keywords:** acceptance, barriers, DiGA, digital health applications, digital therapeutics, discontinuation, mental health, mHealth apps, real-world use, survey

## Abstract

**Background:**

Digital health interventions, including mobile health apps, increasingly complement outpatient mental health care. In Germany, certified digital health applications (DiGA [Digitale Gesundheitsanwendungen]) can be prescribed and reimbursed in the statutory health care system. However, real-world evidence on patient-reported use of DiGA for mental disorders remains limited.

**Objective:**

This study aimed to analyze usage patterns and experiences among DiGA users and expectations among nonusers. Among users, we examined duration of active use, self-reported completion, and experienced reasons for discontinuation; among nonusers, whether DiGA use would be an option and the perceived relevance of hypothetical reasons for discontinuation.

**Methods:**

We conducted an anonymous cross-sectional mixed-mode survey among adults insured by Techniker Krankenkasse, Germany’s largest statutory insurer. Random samples of 2000 individuals per group were invited in April 2024, and data collection ended in September 2024. Users received an activation code for a DiGA authorized for mental disorders. Nonusers had a documented diagnosis for which a DiGA was available but had no prior use. Analyses were descriptive, using available cases, frequencies, and medians with IQRs for ordinal Likert-type items. Actual and intended use were treated as distinct. Exploratory subgroup analyses were conducted.

**Results:**

Overall, 1413 participants were included in the analysis (769 of 2000 invited users; 644 of 2000 invited nonusers), with response rates of 38.5% (95% CI 36.3%-40.6%) and 32.2% (95% CI 30.2%-34.3%), respectively. A total of 840 of 1249 (67.3%; 95% CI 64.6%-69.8%) participants were female. Nonusers were older and more often lived in rural areas. Among users, 487 of 687 (70.9%; 95% CI 67.4%-74.2%) reported active use for at least 5 weeks; 409 of 700 (58.4%; 95% CI 54.7%-62.0%) reported completion of their DiGA program. The proportion of reported completions increased from 111 of 250 (44.4%; 95% CI 38.4%-50.6%) among those aged 18-40 years to 85 of 112 (75.9%; 95% CI 67.2%-82.9%) among those aged 61 years and older. The most frequently endorsed reasons for discontinuation among users reporting noncompletion were lack of motivation (151/272, 55.5%; 95% CI 49.6%-61.3%) and insufficient personalization (114/255, 44.7%; 95% CI 38.7%-50.8%). Among nonusers, the most frequently endorsed hypothetical reasons for discontinuation were mismatch with their condition (431/540, 79.8%; 95% CI 76.2%-83.0%) and technical difficulties (432/545, 79.3%; 95% CI 75.7%-82.5%).

**Conclusions:**

This study provides patient-reported real-world evidence on certified and reimbursable digital mental health interventions. Many users reported weeks of active use, but noncompletion remained common and was more frequent among younger users. Lack of motivation as a frequently reported reason for discontinuation suggests that supporting continued engagement may be important during DiGA use. Beyond Germany, these findings may support implementation of digital mental health interventions in other health care systems.

**International Registered Report Identifier (IRRID):**

RR2-10.2196/56205

## Introduction

Mental health disorders pose a significant public health challenge. In 2021, they accounted for approximately 155.4 million disability-adjusted life years worldwide [1]. Long waiting times between the first contact with a psychotherapist and the start of treatment in Germany highlight the need for complementary approaches in outpatient care [2]. Internationally, digital mental health interventions are increasingly integrated into mental health care [3]. As low-threshold interventions, they may be useful to bridge waiting periods, and supplement outpatient mental health care [4]. The actual benefit of these tools depends on whether and how patients use them in real-world settings. In this context, Germany provides a particularly informative setting for examining real-world use and related challenges, as it was the first country to introduce certified mHealth apps into routine care within a unique regulatory framework.

The German framework for digital health applications (DiGA [Digitale Gesundheitsanwendungen]) was established in 2019 within the Digital Healthcare Act. The first application became available in September 2020 [5,6]. Since then, individuals covered by statutory health insurance (SHI) have access to these certified and reimbursed mHealth apps through a prescription from a physician or psychotherapist or by applying directly to their SHI fund. Similar reimbursement pathways for digital medical devices have since been introduced in other European countries, including France and Belgium [7]. In addition, efforts are underway at the European level to harmonize assessment procedures for digital medical devices [8]. As of October 2026, 30 of the 63 listed DiGA were intended for the treatment of mental disorders. These include applications for depression, anxiety and panic disorders, and stress-related disorders [9]. Despite the large number of mental health DiGA, their overall use in routine care remains limited [6].

Limited use may be related to patients’ expectations and concerns before use. Population-based survey evidence indicates ambivalent attitudes toward digital mental health interventions: while many respondents acknowledged their potential usefulness, 45.8% reported that they would not use such interventions for future psychological problems. Reported concerns included the absence of personal contact, technical difficulties, and issues related to data privacy and security [10]. Once use has been initiated, maintaining engagement poses an additional challenge for mHealth apps and DiGA. Previous research identified usability issues, limited personalization, and difficulties integrating applications into daily routines as barriers to continued engagement [11,12]. In a survey of 1711 insured individuals following a DiGA application, 38.2% reported ending their use before reaching 90 days, the standard prescription period for a DiGA, including 14.8% who ended use within the first month [13]. A real-world study of digital mental health applications outside Germany similarly indicates that early dropout is common and may be associated with user characteristics such as age, education, and symptom burden [14].

However, patient-reported evidence on how mHealth apps for mental disorders in general and DiGA specifically are used in outpatient routine care remains limited. In particular, little is known about actual usage patterns, self-reported completion, and reasons for discontinuation. Evidence is also limited for individuals with relevant mental disorders for which DiGA are available but without prior DiGA approval. It remains unclear whether using one would be an option for them and how they assess them.

Therefore, this study aimed to describe actual and intended use of DiGA for mental disorders by presenting patient-reported use among DiGA users alongside intended use among nonusers. The article presented actual and intended time spent per week using a DiGA and actual and intended locations of use. Among users, the study further examined the overall duration of active use, self-reported completion, and experienced reasons for discontinuation. Among nonusers, it described whether using a DiGA would be an option for them and presented the perceived relevance of hypothetical reasons for discontinuation.

## Methods

### Overview

This manuscript was prepared in accordance with the STROBE (Strengthening the Reporting of Observational Studies in Epidemiology) checklist for cross-sectional studies [15]. The checklist was used to guide the reporting of the study and its design. The completed checklist is reported in Multimedia Appendix 1.

### Study Design and Setting

This study is part of the broader research project “Realizing the Potential of Digital Health Applications (DiGA) in Outpatient Mental Health Care” (DiGAPsy), which is funded by the Federal Joint Committee (funding code 01VSF22029) [16]. The project investigates the integration of DiGA into outpatient mental health care from multiple perspectives [17-19]. This study focuses on the patient survey conducted as part of this project, examining patient-reported attitudes and perceptions.

We conducted an anonymous cross-sectional mixed-mode survey among adults insured by Techniker Krankenkasse (TK), the largest SHI fund in Germany. The study focused on 2 groups: users and nonusers. The index period used for identifying eligible insured individuals was the first and second quarters of 2023. Selected individuals were invited by mail in April 2024, and a reminder letter was sent in May 2024. Data collection was concluded in September 2024.

### Participants, Eligibility Criteria, and Sampling

Eligible participants were adults insured by TK who resided in Germany and were insured by TK during the whole index period and at the time of data extraction. TK identified 2 study groups based on predefined claims-based eligibility criteria.

Users were defined as individuals who had received at least one activation code for a DiGA authorized for the treatment of mental disorders during the index period. Age eligibility was assessed based on the date the activation code was sent, with individuals being eligible if they were aged 18 years or older on that date. For DiGA that could also be used for disorders other than mental disorders, additional DiGA-specific *ICD-10* (*International Statistical Classification of Diseases, Tenth Revision*) criteria were applied to ensure that the approval was related to a mental disorder.

Nonusers were defined as individuals with documented diagnoses for which a DiGA for mental disorders was available, but without any prior DiGA approval. Eligibility was based on predefined DiGA-specific *ICD-10* indications using claims data. Diagnoses had to be documented during the index period or the preceding quarter, and they had to meet the two-quarter criterion. This meant that a diagnosis had to be coded in at least 2 consecutive quarters. Diagnoses were based on outpatient diagnoses, outpatient hospital diagnoses, or main or supplementary inpatient discharge diagnoses. For inpatient main discharge diagnoses, no additional two-quarter criterion was required. Individual suitability for DiGA use, current symptom severity, digital health literacy, and personal living conditions could not be assessed in the claims-based sampling procedure.

Individuals were excluded from both groups if they did not reside in Germany, participated in a selective care program related to mental disorders, were under legal guardianship, had been assigned a long-term care level of at least 2, or had died before data extraction.

After applying the eligibility criteria, TK drew random samples for each study group separately. The target sample size was based on an a priori power calculation for detecting medium-sized group differences with a statistical power of 90% and a 2-sided significance level of α=.05. Under these assumptions, a minimum of 201 participants per group was required. An approximate response rate of 10% was anticipated during study planning, leading to 2000 individuals invited per study group.

### Survey Administration

TK contacted selected individuals by mail. The invitation letter included written study information and the questionnaire materials. Participants could choose between 2 modes of participation. They could complete the paper-based questionnaire using a prepaid return envelope, or they could access an online version of the questionnaire via a QR code. The online survey was implemented using LimeSurvey.

The questionnaire was administered in German. The distribution of survey materials was conducted exclusively by TK and preserved participant anonymity. The research team did not receive directly identifying information. Returned paper questionnaires were sent directly to the University of Duisburg-Essen. Before the survey items, participants received information about the DiGA framework, explaining the general concept of DiGA and its regulatory framework. This section was included to provide a basis for answering the subsequent questions, particularly for nonusers who may not have had prior knowledge about DiGA.

### Variables, Data Sources, and Measurement

All the analytical data were based on responses to a self-report questionnaire. The questionnaire was developed specifically for this study and pretested. It included single-choice items, multiple-choice items, Likert-type items, and optional free-text fields. The responses to those text fields were not included in this analysis. No previously validated instrument was used. The exact wording and response options of the central questionnaire items are provided in Multimedia Appendix 2.

For both study groups, questions related to DiGA use were assessed. Among users, these included actual weekly time spent using their DiGA and locations of use. Among nonusers, the corresponding variables were intended weekly time using a DiGA and intended locations of use. Among users, further variables included reported duration of active use and reported completion of the program. Self-reported completion reflected participants’ own assessment of whether they had completed the DiGA program and was not interpreted as verified completion or use throughout the full 90-day period of prescription. Nonusers were asked whether using a DiGA would be an option for them. Discontinuation-related variables were assessed among users who reported not having completed the DiGA or who were unsure about completion. These users rated experienced reasons for discontinuation. Nonusers were asked to rate hypothetical reasons for discontinuation in a potential use scenario. Although several items used parallel response formats across groups, actual use among users and hypothetical assessments among nonusers were treated as conceptually distinct. Optional free-text fields allowed participants to provide additional experienced or hypothetical reasons for discontinuation; these responses are reported in Multimedia Appendix 3.

Subgroup variables included age group, gender, place of residence, and education level. The study did not assess clinical outcomes or DiGA effectiveness. The exploratory subgroup analyses were unadjusted and aimed to describe patterns within the sample, rather than attempting to estimate causal effects.

### Bias

Several design measures were used to reduce potential bias. Random samples were used to reduce selection bias at the sampling stage, and a reminder letter was sent to increase participation. The anonymous survey design was intended to reduce socially desirable responding. Nevertheless, given that participation was voluntary and all participants were sampled from one SHI fund, issues of response bias and limited generalizability may be pertinent. Paper-based and web-based questionnaires contained equivalent items and response options to limit potential mode effects, and the questionnaire was pretested for comprehensibility and feasibility.

### Handling of Variables and Data Cleaning

Most variables were nominal or ordinal. Some sociodemographic variables were grouped for descriptive and exploratory subgroup analyses. Age was grouped into 18-40 years, 41-60 years, and 61 years or older. Place of residence was grouped into rural, middle, and urban areas. Because the number of participants identifying as diverse was too small for separate subgroup reporting, gender-based subgroup analyses were restricted to male and female participants. Education level was grouped into no completed vocational training or academic degree, vocational education, and university or university of applied sciences degree (Table S1 in Multimedia Appendix 2). Actual and intended weekly time spent using a DiGA were interpreted according to the predefined response categories. The answers regarding actual and intended locations of use were multiple-response variables. Likert-type items on experienced or hypothetical reasons for discontinuation were interpreted as ordinal variables. The middle response category was “partly applies, partly does not apply” and was interpreted as a valid response category.

Data preparation and statistical analyses were conducted with R version 4.4.1. Due to differences in data formats between paper-based and online responses, both datasets were cleaned separately before being merged. All data cleaning steps were performed before further analyses. Online questionnaires were excluded if participants accessed the survey but did not proceed with the questionnaire. Paper-based and online questionnaires were classified as invalid and excluded if the first survey question was not answered.

The questionnaire content was designed to be equivalent across both modes. For single-choice items, responses were treated as invalid if more than one response option was selected. For Likert-type items, responses were treated as invalid if more than one scale point was marked. Filter-dependent items were checked for consistency. Responses to subsequent items were treated as invalid if the respective filter criterion was not met. Optional free-text fields were not systematically analyzed.

### Missing Data

Analyses were conducted using an available-case approach. Participants were included in each analysis if they provided a valid response to the respective item. Therefore, valid responses vary between variables. Structural missing values resulting from questionnaire routing were not treated as missing data. Detailed information on valid and missing responses is provided in Tables S3-S6 in Multimedia Appendix 2.

### Data Analysis

Categorical variables were reported as absolute and relative frequencies using valid denominators. Ordinal Likert-type items were additionally summarized using medians and IQRs. Both user and nonuser assessments were presented side by side and interpreted descriptively. Sociodemographic characteristics of users and nonusers were presented descriptively and were not formally compared using inferential tests. Given the descriptive aim of the study, no multiple imputation was performed.

Exploratory subgroup analyses were conducted according to the level of measurement. Chi-square tests were used for categorical variables. Phi was used as a measure of association for 2×2 tables and Cramer V for larger contingency tables. Ordinal variables and Likert-type items were analyzed using Mann-Whitney *U* tests for 2-group subgroup comparisons and Kruskal-Wallis tests for comparisons across more than 2 groups. Where applicable, rank-biserial correlation or Kendall τ-c were reported as measures of association. All tests were 2-sided with a significance level of α=.05 (Table S2 in Multimedia Appendix 2).

### Ethical Considerations

The study was approved by the Ethics Committee of the Medical Faculty of the University of Duisburg-Essen (reference number 23-11209-BO) and was conducted in accordance with the Declaration of Helsinki. Participation was voluntary. Participants received written study information stating that informed consent was provided by completing and returning or submitting the questionnaire. Due to the anonymous survey design, no separate signed informed consent was collected.

Participant anonymity was ensured by the recruitment procedure and data collection process. Survey materials were distributed exclusively by TK, completed paper questionnaires were returned directly to the University of Duisburg-Essen, and online responses were collected without personal identifiers. Participants did not receive compensation. No images or other visual materials containing identifiable participant information are included in the manuscript or supplementary materials.

## Results

### Overview

A total of 1413 questionnaires were included in the analysis. Of the 2000 invited nonusers, 644 valid questionnaires were returned, equating to a response rate of 32.2%. Of users, 769 of the 2000 contacted individuals participated, resulting in a response rate of 38.5%. Further information can be found in Figure 1.

**Figure 1:**
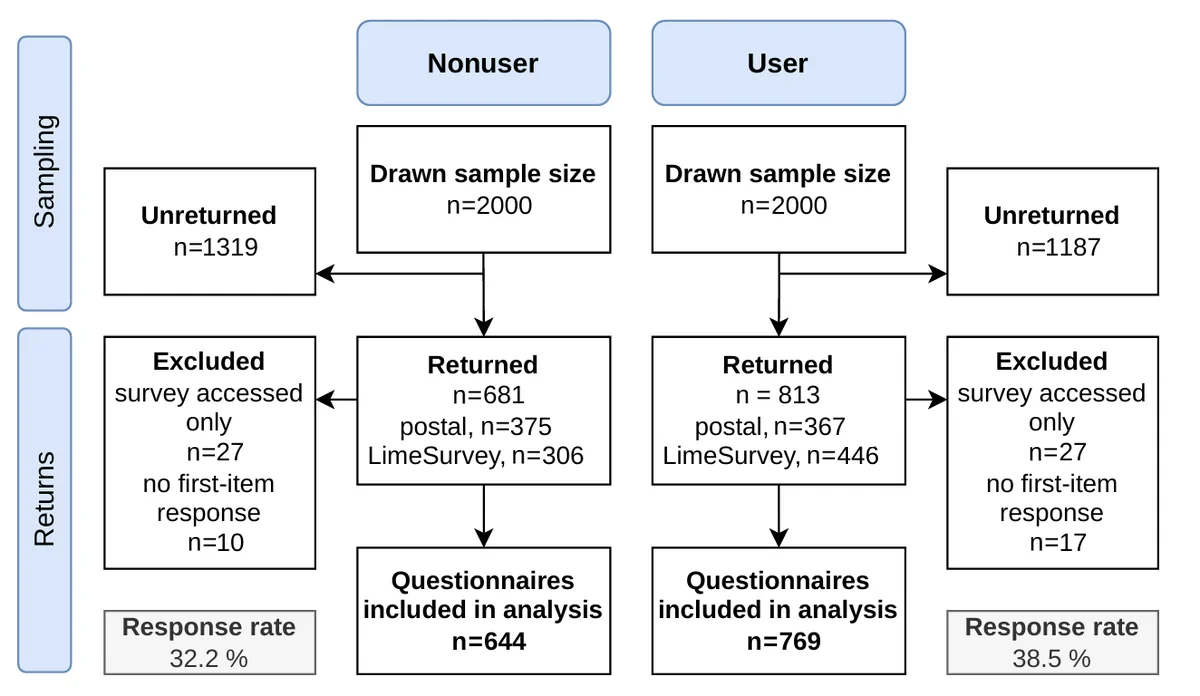
Participant flow by study group in a cross-sectional survey of digital health applications use for mental disorders among adults insured by Techniker Krankenkasse, 2024.

Among users, Little's missing completely at random (MCAR) test did not reject the MCAR hypothesis for sociodemographic variables (*χ*^2^_14_=12.6; *P*=.56) or general questionnaire variables (*χ*^2^_31_=38.8; *P*=.16), whereas the MCAR hypothesis was rejected for experienced reasons for discontinuation (*χ*^2^_380_=467.0; *P*=.001). Among nonusers, the MCAR hypothesis was not rejected for sociodemographic variables (*χ*^2^_18_=12.6; *P*=.82) or hypothetical reasons for discontinuation (*χ*^2^_241_=258.0; *P*=.21), whereas it was rejected for general questionnaire variables (*χ*^2^_17_=94.8; *P*<.001). Detailed information on the included variables and missingness patterns is provided in Table S3 in Multimedia Appendix 2.

Participant characteristics are presented in Table 1. Among participants, 840/1249 (67.3%) identified as female. The proportion was higher among users (489/678, 72.1%) than among nonusers (351/571, 61.5%). With regard to age, the largest proportion of participants (538/1261, 42.7%) were between 41 and 60 years of age. Users tended to be younger, with a higher percentage falling within the 18-40 years age group (users: 254/682, 37.2%; nonusers: 102/579, 17.6%). In contrast, participants aged 61 years or older were more strongly represented among nonusers (users: 114/682, 16.7%; nonusers: 253/579, 43.7%).

In terms of education, 52.5% (621/1183) of the participants reported having completed vocational education and training, and 42.4% (502/1183) had obtained a university degree. Users slightly more often reported a university degree (users: 293/654, 44.8%; nonusers: 209/529, 39.5%), whereas vocational education and training was more common among nonusers (users: 329/654, 50.3%; nonusers: 292/529, 55.2%). The proportion of participants without any completed qualification was low in both groups (users: 32/654, 4.9%; nonusers: 28/529, 5.3%). Regarding place of residence, many participants lived in urban areas (524/1259, 41.6%). Users were more frequently located in urban areas (users: 315/682, 46.2%; nonusers: 209/576, 36.3%), whereas nonusers were more likely to reside in rural areas (users: 239/682, 35%; nonusers: 241/576, 41.8%). Sociodemographic variables were not completed by all participants (Table S4 in Multimedia Appendix 2).

**Table 1:** Sociodemographic characteristics by study group among participants in a cross-sectional survey on digital health applications for mental disorders, Germany, 2024.

Characteristics			Nonusers (n=644), n (%)		Users (n=769), n (%)		Combined (n=1413), n (%)		Reference population^a^ (n=10,022,017), n (%)
Gender									
	Male	220 (38.5)		189 (27.9)		409 (32.7)		4,991,632 (49.81)	
	Female	351 (61.5)		489 (72.1)		840 (67.3)		5,030,385 (50.19)	
Age (years)									
	18-40	102 (17.6)		254 (37.2)		356 (28.2)		3,888,246 (38.8)	
	41-60	224 (38.6)		314 (46.0)		538 (42.7)		3,383,111 (33.76)	
	≥61	253 (43.7)		114 (16.7)		367 (29.1)		2,750,660 (27.45)	
Education									
	No completed education	28 (5.3)		32 (4.9)		60 (5.1)		—^b^	
	Vocational education and training	292 (55.2)		329 (50.3)		621 (52.5)		—	
	University degree	209 (39.5)		293 (44.8)		502 (42.4)		—	
Place of residence									
	Rural/small town (<20,000)	241 (41.8)		239 (35.0)		480 (38.2)		—	
	Medium-sized city (20,000-99,999)	126 (21.9)		128 (18.8)		254 (20.2)		—	
	Urban/large city (≥100,000)	209 (36.3)		315 (46.2)		524 (41.7)		—	

^a^Population covered by TK in the first quarter of 2024.

^b^Not available.

### Usage and Attitudes Toward (Hypothetical) Usage

Among nonusers, responses were nearly evenly divided between considering DiGA use an option and being unsure whether it would be an option, whereas only a small proportion reported that DiGA use would not be an option. Among nonusers aged 18-40 years, 59.6% (59/99) reported that DiGA use would be an option, compared with 46.8% (103/220) among those aged 41-60 years and 35.8% (88/246) among participating nonusers aged 61 years or older (Tables S7 and S8 in Multimedia Appendix 2).

Among nonusers, 257 of 589 (43.6%, 95% CI 39.7%-47.7%) reported that DiGA use was an option for them, whereas 73 of 589 (12.4%, 95% CI 10.0%-15.3%) reported that it was not an option and 259 of 589 (44.0%, 95% CI 40.0%-48.0%) were unsure.

### Actual and Intended Weekly Time Spent Using a DiGA

Among users, the most frequently reported weekly use category was 30 to 60 minutes (307/693, 44.3%). This was likewise the most frequently reported weekly use category among nonusers (236/553, 42.7%). However, less than 30 minutes was reported more often as intended use among nonusers than as actual use among users (212/553, 38.3% vs 147/693, 21.2%; Table 2).

In terms of the location of use, 701/704 (99.6%, 95% CI 98.8%-99.9%) of users reported that they used their DiGA predominantly at home. Use while commuting was reported by 97/704 (13.8%, 95% CI 11.4%-16.5%) and use at work by 45/704 (6.4%, 95% CI 4.8%-8.4%). Among nonusers, 522/562 (92.9%, 95% CI 90.5%-94.7%) intended to use a DiGA at home, while 160/562 (28.5%, 95% CI 24.9%-32.3%) intended to use one while commuting and 78/562 (13.9%, 95% CI 11.3%-17.0%) intended to use one at work. Multiple responses were permitted, and percentages refer to participants with valid responses.

Among users, 9 to 12 weeks was the most frequently reported duration of active DiGA use (199/687, 29%), and 487 of 687 (70.9%) reported active use for at least 5 weeks (Table 3). The proportion reporting less than 5 weeks of active use was higher among users aged 18-40 years (73/245, 29.8%) than among those aged 41-60 years (44/302, 14.6%) or ≥61 years (13/110, 11.8%; Tables S9 and S10 in Multimedia Appendix 2).

**Table 2:** Actual weekly time spent using digital health applications (DiGA) among users and intended weekly time among nonusers in a cross-sectional survey on DiGA for mental disorders.

Weekly time (minutes)	Nonusers: intended use (n=553), n (%; 95% CI)	Users: actual use (n=693), n (%; 95% CI)
Less than 30	212 (38.3; 34.4-42.5)	147 (21.2; 18.3-24.4)
30-60	236 (42.7; 38.6-46.8)	307 (44.3; 40.6-48.0)
60-90	76 (13.7; 11.1-16.9)	136 (19.6; 16.8-22.7)
More than 90	29 (5.2; 3.7-7.4)	53 (7.6; 5.9-9.9)
Unsure	—^a^	50 (7.2; 5.5-9.4)

^a^Not available.

**Table 3:** Reported duration of active digital health applications (DiGA) use in weeks among users in a cross-sectional survey on DiGA for mental disorders.

Use period in weeks	Users (n=687), n (%; 95% CI)
Less than 1	13 (1.9; 1.1-3.2)
1-4	129 (18.8; 16.0-21.9)
5-8	155 (22.6; 19.6-25.8)
9-12	199 (29.0; 25.7-32.5)
More than 12	133 (19.4; 16.6-22.5)
Unsure	58 (8.4; 6.6-10.8)

### Reported Completion

Among users, 409 of 700 (58.4%) reported having completed their DiGA program, while 259 of 700 (37%) reported noncompletion (Table 4). Reported completion was more common in older age groups, increasing from 111 of 250 (44.4%) among users aged 18-40 years to 203 of 306 (66.3%) among those aged 41-60 years and 85 of 112 (75.9%) among those aged ≥61 years. In addition, of the users who reported completing their DiGA program and provided valid information on the duration of their active use, 266/396 (67.2%) reported active use for at least 9 weeks. This compares with 49/254 (19.3%) users who reported not completing their DiGA program (Tables S11-S13 in Multimedia Appendix 2).

**Table 4:** Self-reported completion status among users in a cross-sectional survey on digital health applications for mental disorders.

Completion status	Users (n=700), n (%; 95% CI)
Completed	409 (58.4; 54.7-62.0)
Not completed	259 (37.0; 33.5-40.6)
Unsure	32 (4.6; 3.3-6.4)

### Experienced and Hypothetical Reasons for Premature Discontinuation

#### Overview

Tables 5 and 6 show an overview of self-reported reasons for discontinuation among users and hypothetical reasons for discontinuation among nonusers. Respondents rated statements representing (hypothetical) reasons for discontinuation using a 5-point Likert scale ranging from “strongly disagree” to “strongly agree.”

**Table 5:** Experienced reasons for discontinuation among users who reported noncompletion or were unsure about completion in a cross-sectional survey on digital health applications (DiGA) for mental disorders.

I did not continue using the DiGA because...	Valid (n=289), n	Median (IQR)	Strongly disagree, n (%)	Rather disagree, n (%)	Partly applies, partly does not apply, n (%)	Rather agree, n (%)	Strongly agree, n (%)
The effect I had desired for had already occurred.	261	1 (1-3)	140 (53.6)	28 (10.7)	45 (17.2)	31 (11.9)	17 (6.5)
No effectiveness was perceived.	259	3 (1-4)	72 (27.8)	35 (13.5)	51 (19.7)	53 (20.5)	48 (18.5)
The DiGA was not compatible with my daily routines.	258	3 (1-4)	72 (27.9)	32 (12.4)	73 (28.3)	51 (19.8)	30 (11.6)
The DiGA had technical difficulties.	267	1 (1-1)	226 (84.6)	12 (4.5)	12 (4.5)	9 (3.4)	8 (3)
I got bored during using it.	263	3 (1-4)	77 (29.3)	29 (11)	70 (26.6)	51 (19.4)	36 (13.7)
the DiGA could not be sufficiently personalized.	255	3 (2-4)	56 (22)	31 (12.2)	54 (21.2)	63 (24.7)	51 (20)
I was going through an acute phase of my disorder.	259	3 (1-4)	109 (42.1)	13 (5)	24 (9.3)	49 (18.9)	64 (24.7)
I lacked motivation to use it.	272	4 (3-5)	37 (13.6)	15 (5.5)	69 (25.4)	77 (28.3)	74 (27.2)
The DiGA did not match my condition.	242	2 (1-3)	118 (48.8)	41 (16.9)	47 (19.4)	14 (5.8)	22 (9.1)
A DiGA in general is not suitable for me.	244	1 (1-3)	130 (53.3)	43 (17.6)	38 (15.6)	14 (5.7)	19 (7.8)

**Table 6:** Perceived relevance of hypothetical reasons for discontinuation among nonusers in a cross-sectional survey on digital health applications (DiGA) for mental disorders.

I would not continue using a DiGA, if...	Valid (n=561), n	Median (IQR)	Strongly disagree, n (%)	Rather disagree, n (%)	Partly applies, partly does not apply, n (%)	Rather agree, n (%)	Strongly agree, n (%)
The effect I had desired had already occurred.	528	3 (3-4)	49 (9.3)	81 (15.3)	157 (29.7)	111 (21)	130 (24.6)
No improvements were perceived from using it.	545	4 (3-5)	26 (4.8)	27 (5)	96 (17.6)	149 (27.3)	247 (45.3)
It was not compatible with my daily routines.	545	4 (3-5)	34 (6.2)	38 (7)	90 (16.5)	129 (23.7)	254 (46.6)
It had technical difficulties.	545	5 (4-5)	21 (3.9)	32 (5.9)	60 (11)	117 (21.5)	315 (57.8)
I got bored while using it.	535	4 (3-5)	55 (10.3)	73 (13.6)	134 (25)	124 (23.2)	149 (27.9)
It could not be sufficiently personalized.	536	4 (3-5)	21 (3.9)	37 (6.9)	130 (24.3)	164 (30.6)	184 (34.3)
I was going through an acute phase of my disorder.	535	3 (2-4)	75 (14)	87 (16.3)	169 (31.6)	101 (18.9)	103 (19.3)
I lacked motivation to use it.	544	4 (3-4)	55 (10.1)	58 (10.7)	155 (28.5)	142 (26.1)	134 (24.6)
It did not match my condition.	540	5 (4-5)	31 (5.7)	20 (3.7)	58 (10.7)	103 (19.1)	328 (60.7)
A DiGA in general is not suitable for me.	542	5 (3-5)	44 (8.1)	34 (6.3)	83 (15.3)	95 (17.5)	286 (52.8)

#### Reported Reasons for Premature Discontinuation

The most commonly agreed reason for discontinuation among users who reported not having completed their DiGA program or were unsure about completion was lack of motivation. A total of 55.5% (151/272) rather agreed or strongly agreed that they did not continue because they lacked motivation. Other commonly endorsed reasons were insufficient personalization of their DiGA (114/255, 44.7%) and going through an acute phase of their disorder (113/259, 43.6%), as well as a perceived lack of effectiveness (101/259, 39%). In contrast, several reasons were rarely agreed upon. Technical difficulties were reported by 6.4% (17/267), and 14.9% (36/242) rather or strongly agreed that the reason for premature discontinuation was that the DiGA did not match their condition.

Exploratory subgroup analyses suggested that lack of motivation and boredom during use were more frequently indicated by younger users. Among those aged 18-40 years, 67.7% (90/133) rather agreed or strongly agreed that they lacked motivation to continue using the DiGA, compared with 42.9% (42/98) among users aged 41-60 years and 28.6% (6/21) among users aged 61 years or older. A similar pattern was observed for boredom during use, which was endorsed by 42.2% (54/128) of users aged 18-40 years, compared with 23.7% (23/97) and 15% (3/20) in the older age groups, respectively. A gender-related pattern was observed regarding insufficient personalization. Insufficient personalization was cited as a reason for discontinuation by 48.2% (81/168) of female users, compared to 33.3% (24/72) of male users (Tables S14-19 in Multimedia Appendix 2). A total of 69 users provided additional answers in the optional free-text field. These responses are shown in Table S1 in Multimedia Appendix 3.

#### Hypothetical Reasons for Discontinuation

The most frequently endorsed hypothetical reason for premature discontinuation was mismatch between the DiGA and the individual’s condition. A total of 79.8% (431/540) of respondents rather agreed or strongly agreed with this statement, followed by technical difficulties (432/545, 79.3%). Perceived lack of effectiveness was endorsed by 72.7% (396/545) of respondents, while 70.3% (383/545) reported that incompatibility with daily routines would be a relevant reason for discontinuation. Similarly, 70.3% (381/542) either rather agreed or strongly agreed that they would discontinue use if a DiGA was generally not suitable for them. Other hypothetical reasons were agreed upon less frequently, but still by a large proportion of nonusers. These were insufficient personalization (348/536, 64.9%), boredom during use (273/535, 51%), and lack of motivation (276/544, 50.7%). The lowest levels of agreement were observed for the desired effect having already occurred (241/528, 45.6%) and for the disorder being in an acute phase (204/535, 38.1%).

Exploratory subgroup analyses suggested that age-related patterns were also present among nonusers. Incompatibility with daily routines was endorsed by 78.9% (75/95) of individuals aged 18-40 years and 76.1% (159/209) of individuals aged 41-60 years, compared to 62.3% (139/223) of those aged 61 years or older. A similar pattern was observed for mismatch with the individual's condition, which was agreed upon by 87.2% (82/94) of nonusers aged 18-40 years, 83.3% (174/209) of those aged 41-60 years, and 74.4% (163/219) of those aged 61 years or older (Tables S20-23 in Multimedia Appendix 2). A total of 17 nonusers provided additional answers in the optional free-text field. These responses are shown in Table S2 in Multimedia Appendix 3.

## Discussion

### Principal Findings

This study provided patient-reported data on the actual and intended use of DiGA for mental disorders in Germany by examining data from 769 users and 644 nonusers. Overall, users and nonusers differed in their sociodemographic profiles. Users tended to be younger, more highly educated, and more likely to live in urban areas. Among nonusers, DiGA use was frequently considered an option. Users most often reported weekly use of 30 to 60 minutes, and both actual and intended use were mainly home-based. About 71% (487/687) of users reported active use for at least 5 weeks. In addition, more than half of users reported completion, and older participants reported completion more often than younger participants. The study also identified experienced reasons for discontinuation among users and the perceived relevance of hypothetical reasons among nonusers.

It is noteworthy that a rural place of residence is more common among nonusers. Limited access to mental health care may increase the potential of digital interventions in rural areas [20]. The higher number of participating nonusers from rural areas suggests that this potential may not yet have been fully realized. One reason for this may be an older demographic of rural populations [20]. Because no clear place-of-residence–related patterns were observed in the assessed outcomes, the higher proportion of rural nonusers may partly reflect sociodemographic differences, particularly the older age structure of rural populations, rather than systematic differences in attitudes toward DiGA.

One aim of this study was to investigate reported usage patterns. The majority of participating users indicated that they had used their DiGA for multiple weeks. Nevertheless, the number of users who reported noncompletion shows that a significant proportion of users do not finish their DiGA program and that this proportion in routine care is higher than in many approval studies, although the different measures of discontinuation limit direct comparability [21]. Our pattern with about 58.4% (409/700) reporting completion is somewhat consistent with survey evidence from Germany’s second biggest SHI fund, where 53.0% of users reported using their DiGA for the full 90-day period [13]. Notably, reported completion cannot be equated with use throughout the standard prescription period of 90 days, but not all DiGA programs necessarily require active use for this long to complete; some are designed for shorter durations [22,23]. In our cross-tabulation, users reporting completion generally reported longer periods of active use, although only 27.8% (110/396) reported active use for more than 12 weeks. This shows that self-reported completion and duration of active use capture related but distinct aspects of DiGA use: participants may have considered their program completed without using the DiGA throughout the full standard prescription period. Reported completion was more common among older than younger users in our study. Analogous age-related patterns have been reported in the context of other mental health applications. For instance, an examination of an insomnia app revealed that younger age was associated with premature discontinuation [14]. Higher rates of discontinuation have also been observed in other types of interventions for mental disorders, including medication and outpatient psychiatric treatment [4,24]. Further research is therefore required to identify the underlying factors of the observed age-related differences in self-reported completion.

The discontinuation-related findings need to be interpreted in light of the different perspectives assessed in the 2 study groups. Users reported reasons for discontinuation based on their actual DiGA use, whereas nonusers rated the perceived relevance of hypothetical reasons in a potential use scenario. Technical difficulties, a mismatch with one’s condition, and the general unsuitability of DiGA were among the most frequently endorsed hypothetical reasons for discontinuation of nonusers. This is consistent with acceptance frameworks, which suggest that perceived usefulness and concerns about usability shape evaluations of digital health interventions [25]. Our findings also align with recurring themes reported in literature on digital mental health interventions. A scoping review by Giebel et al [11] highlights technical issues and poor contextual fit as frequent barriers that can hinder uptake. A lack of motivation appeared to be particularly relevant among users who reported premature discontinuation. In the context of mental disorders, a lack of motivation may be related to symptoms, particularly in cases of depression, where low energy levels and reduced motivation can impede the continuation of digital interventions [11,26]. Our findings are consistent with previous research on digital mental health interventions, which also identify motivational factors as an important determinant for continued use [12].

The high proportions of agreement assigned to hypothetical discontinuation reasons among nonusers suggest that information provided prior to DiGA use may need to address topics such as technical reliability and perceived fit. In addition to this, a voluntary assessment before use could be considered to identify use-relevant characteristics. These implications are consistent with the German debate on the implementation of DiGA, which also emphasizes the importance of clear information and trust [27,28]. From a patient-centered perspective, approaches aimed at supporting continued engagement may be relevant in the design and implementation of DiGA. In this context, blended-care approaches or forms of human support may be particularly relevant, as evidence suggests that guided digital mental health interventions may improve effectiveness when compared with unguided approaches [29]. Gamification approaches could also be explored as a potential strategy to support engagement, given that gamified digital mental health interventions are considered a promising approach [30]. Further research is needed to identify the factors underlying the age-related differences observed in this study and to examine whether particular engagement strategies are beneficial for different user groups.

### Strengths and Limitations

A significant strength of this study is its 2-group design, which includes both individuals with and without prior DiGA approval. This design enables a side-by-side presentation of experienced reasons among users and hypothetical reasons among nonusers. Furthermore, the study is based on a large sample, enabling detailed descriptive analyses and exploratory subgroup analyses.

Several limitations should be considered. All results are based on self-reported data and are therefore subject to recall bias and social desirability bias. Despite the anonymous design, social desirability may have favored reports of more sustained use and completion and thereby contributed to an underestimation of noncompletion, whereas the direction of recall bias is less predictable. The cross-sectional design does not permit causal interpretations. In addition, the exploratory subgroup analyses were unadjusted. Consequently, the observed subgroup differences may be subject to confounding by measured or unmeasured characteristics that differ between groups and should not be interpreted as independent effects. Moreover, users reported on actual experiences retrospectively, whereas nonusers responded to hypothetical scenarios. These perspectives are therefore conceptually distinct and should not be interpreted as directly equivalent measurements.

Further limitations relate to the definition of the nonuser group and the generalizability of the findings. The receipt of an activation code does not confirm actual DiGA use. Therefore, some individuals classified as users may not have actually started their DiGA program. Nonusers were identified based on documented diagnoses in areas for which DiGA were available, and on the absence of DiGA approval. However, it was not possible to assess individual suitability for DiGA use, for example, based on current symptom severity, digital health literacy, or personal living conditions. Therefore, not all nonusers can be assumed to be potential DiGA users. Missing data occurred for several variables, particularly sociodemographic variables placed toward the end of the questionnaire, which may limit the completeness of sample characterization and exploratory subgroup analyses. Optional free-text fields were not systematically analyzed, so the findings primarily reflect predefined questionnaire items. Finally, the study was conducted within the German DiGA context and among insured individuals from one SHI fund. The relatively high proportion of participants with a university degree may indicate higher digital literacy than in the general population, which could have led to an underestimation of technical barriers and an overestimation of willingness or ability to use DiGA.

### Conclusion

In conclusion, this study adds real-world evidence on how DiGA for mental disorders are used and evaluated in outpatient mental health care, differentiating between the experiences of users and hypothetical assessments of nonusers. The findings suggest that a successful implementation needs to address different perspectives. Clear information on suitability, technical requirements, and what users can expect from DiGA use may support informed decisions before initiation, whereas human support and engagement-oriented design approaches may warrant consideration for supporting continued engagement. These implications may also be relevant to the implementation of digital mental health interventions beyond the German DiGA framework.

## Appendix

Multimedia Appendix 1 STROBE checklist.

Multimedia Appendix 2 Methods and subgroup analyses.

Multimedia Appendix 3 Free-text responses on additional reasons for discontinuation.

## Abbreviations

DiGA: digital health applications (Digitale Gesundheitsanwendungen)
DiGAPsy: Realizing the Potential of Digital Health Applications (DiGA) in Outpatient Mental Health Care
ICD-10: International Statistical Classification of Diseases, Tenth Revision
MCAR: missing completely at random
mHealth: mobile health
SHI: statutory health insurance
STROBE: Strengthening the Reporting of Observational Studies in Epidemiology
TK: Techniker Krankenkasse

## Data Availability

The datasets generated and analyzed during this study are not publicly available due to data protection requirements. The data cannot be made available upon request because data sharing is not permitted under the applicable data protection requirements.

## References

[1] Fan Y, Fan A, Yang Z, Fan D (2025). Global burden of mental disorders in 204 countries and territories, 1990-2021: results from the global burden of disease study 2021. BMC Psychiatry.

[2] Schlierenkamp S, Werner S, Schlesiger P, Friedrich L, Abels C, Höfer K, Best D, Lubisch B, Hentschel G, Weigel F, Wieczorek D, Marschall U, Schaff C, Meisel B, Timmermann H, Neumann A, Walendzik A, Wasem J, Neusser S (2025). Treatment pathways in the outpatient care of patients with depression - an analysis of statutory health insurance claims data. Psychiatr Prax.

[3] Hall CL, Gómez Bergin AD, Rennick-Egglestone S (2024). Research into digital health intervention for mental health: 25-year retrospective on the ethical and legal challenges. J Med Internet Res.

[4] Huang S, Wang Y, Li G, Hall BJ, Nyman TJ (2024). Digital mental health interventions for alleviating depression and anxiety during psychotherapy waiting lists: systematic review. JMIR Ment Health.

[5] Bundesministerium für Gesundheit (2019). Driving the digital transformation of Germany's healthcare system for the good of patients 2019. Bundesministerium für Gesundhe.

[6] (2026). DiGA-Bericht des GKV-Spitzenverbandes -2025: bericht über die inanspruchnahme und entwicklung der versorgung mit Digitalen Gesundheitsanwendungen. GKV-Spitzenverband.

[7] van Kessel R, Srivastava D, Kyriopoulos I, Monti G, Novillo-Ortiz D, Milman R, Zhang-Czabanowski WW, Nasi G, Stern AD, Wharton G, Mossialos E (2023). Digital health reimbursement strategies of 8 European countries and Israel: scoping review and policy mapping. JMIR Mhealth Uhealth.

[8] Tarricone R, Petracca F, Weller H-M (2024). "Towards harmonizing assessment and reimbursement of digital medical devices in the EU through mutual learning". NPJ Digit Med.

[9] DiGA-Verzeichnis. BfArM.

[10] Steubl LS, Büscher R, Sander LB, Baumel A, Barck K, Sachser C, Fegert JM, Brähler E, Baumeister H, Domhardt M (2025). Attitudes towards digital health interventions in Germany: findings from a population-based representative survey. Clin Psychol Eur.

[11] Giebel GD, Speckemeier C, Abels C, Plescher F, Börchers K, Wasem J, Blase N, Neusser S (2023). Problems and barriers related to the use of digital health applications: scoping review. J Med Internet Res.

[12] Borghouts J, Eikey E, Mark G, De Leon C, Schueller SM, Schneider M, Stadnick N, Zheng K, Mukamel D, Sorkin DH (2021). Barriers to and facilitators of user engagement with digital mental health interventions: systematic review. J Med Internet Res.

[13] Grobe T, Weller L, Braun A, Szecsenyi J BARMER Arztreport 2024. BARMER Institut für Gesundheitssystemforschung.

[14] Sanchez Ortuño MM, Pecune F, Coelho J, Micoulaud-Franchi JA, Salles N, Auriacombe M, Serre F, Levavasseur Y, De Sevin E, Sagaspe P, Philip P (2025). Determinants of dropout from a virtual agent-based app for insomnia management in a self-selected sample of users with insomnia symptoms: longitudinal study. JMIR Ment Health.

[15] Cuschieri S (2019). The STROBE guidelines. Saudi J Anaesth.

[16] Höfer K, Plescher F, Schlierenkamp S, Solar S, Neusser S, Schneider U, Best D, Wasem J, Abels C, Bußmann A (2024). mHealth apps in German outpatient mental health care: protocol for a mixed methods approach. JMIR Res Protoc.

[17] Volland C, Plescher F, Raszke P, Friedrich L, Speckemeier C, Schlierenkamp S, Minor M, Abels C, Höfer K, Wasem J, Katzmann J, Schneider U, Wadeck A, Zander S, Bußmann A (2026). Prescriber's preferences for digital health applications in mental health care: cross-sectional best-worst scaling study of general practitioners and psychotherapists in Germany. J Med Internet Res.

[18] Plescher F, Höfer K, Wasem J, Bußmann A, Solar S, Minor M, Schlierenkamp S, Best D, Katzmann J, Maaß E, Schneider U, Wadeck A, Zander S, Abels C (2025). Concepts for the integration and implementation of mHealth apps for patients with mental disorders: scoping review. J Med Internet Res.

[19] Giebel G, Höfer K, Raszke P, Wasem J, Bußmann A, Schlierenkamp S, Minor M, Volland C, Schneider U, Maaß E, Katzmann J, Abels C (2026). Access to digital health applications - prescription by physicians vs patient requests made directly to health insurance companies: interviews with experts. Gesundheitswesen.

[20] Watanabe J, Teraura H, Nakamura A, Kotani K (2023). Telemental health in rural areas: a systematic review. J Rural Med.

[21] Sippli K, Deckert S, Schmitt J, Scheibe M (2025). Healthcare effects and evidence robustness of reimbursable digital health applications in Germany: a systematic review. NPJ Digit Med.

[22] Schuffelen J, Maurer LF, Gieselmann A (2026). The effects of digital cognitive behavioral therapy for insomnia in chronic pain: a randomized controlled trial. Psychother Psychosom.

[23] Mäder M, Timpel P, Schönfelder T, Militzer-Horstmann C, Scheibe S, Heinrich R, Häckl D (2023). Evidence requirements of permanently listed digital health applications (DiGA) and their implementation in the German DiGA directory: an analysis. BMC Health Serv Res.

[24] Ishizuka K, Ishiguro T, Kawaguchi D, Nomura N, Inada T (2023). Factors predicting early treatment discontinuation among depressed patients of working age in a psychiatric outpatient practice. Psychiatry Res Commun.

[25] Philippi P, Baumeister H, Apolinário-Hagen J, Ebert DD, Hennemann S, Kott L, Lin J, Messner E, Terhorst Y (2021). Acceptance towards digital health interventions - model validation and further development of the unified theory of acceptance and use of technology. Internet Interv.

[26] Robert Koch-Institut (2025). Depressive und Angstsymptomatik bei Erwachsenen in Deutschland: Ergebnisse aus dem Panel „Gesundheit in Deutschland“ 2024. Gesundheitsberichterstattung.

[27] Engesser D, Singer S, Kemmerer P, Dotzauer L, Salm S, Scheibe S, Mergenthal K, Voigt K, Braun B, Imhorst E, Kobes J, Lausmann K, Marx C, Petermann-Meyer A, Reefschläger G, Wirp B, Stalujanis E (2025). Digitale Gesundheitsanwendungen für psychische erkrankungen – für wen und wann (nicht)?. Z Klin Psychol Psychother.

[28] Stalujanis E, Kemmerer P, Engesser D, Dotzauer L, Salm S, Scheibe S, Mergenthal K, Voigt K, Singer S (2026). Zwischen Hoffnung, Hype und Hürden: Digitale Gesundheitsanwendungen für psychische Störungen. Psychotherapie.

[29] Thompson EM, Destree L, Albertella L, Fontenelle LF (2021). Internet-based acceptance and commitment therapy: a transdiagnostic systematic review and meta-analysis for mental health outcomes. Behav Ther.

[30] Aschentrup L, Steimer PA, Dadaczynski K, Mc Call T, Fischer F, Wrona KJ (2024). Effectiveness of gamified digital interventions in mental health prevention and health promotion among adults: a scoping review. BMC Public Health.

