# Supplementary material for: Digital Health Applications for Mental Disorders in Germany Among Users and Nonusers on Actual and Intended Use, Self-Reported Completion, and Reasons for Discontinuation: Cross-Sectional Survey
Source: J Med Internet Res. 2026 Oct 29;28:e99464. doi: 10.2196/99464 (PMC13760326; doi:10.2196/99464)
Supplement: Supplementary file 1 [file jmir_v28i1e99464_app1.pdf]

Multimedia Appendix 1: Strengthening the reporting of observational studies in epidemiology (STROBE)

STROBE Statement - Checklist of items that should be included in reports of ***cross-sectional studies***

	Item No	Recommendation	Location in article
**Title and abstract**	1	(*a*) Indicate the study's design with a commonly used term in the title or the abstract	Title, Abstract
		(*b*) Provide in the abstract an informative and balanced summary of what was done and what was found	Abstract
**Introduction**			
Background/rationale	2	Explain the scientific background and rationale for the investigation being reported	Introduction
Objectives	3	State specific objectives, including any prespecified hypotheses	Introduction: Final paragraph
**Methods**			
Study design	4	Present key elements of study design early in the paper	Methods: Study Design and Setting
Setting	5	Describe the setting, locations, and relevant dates, including periods of recruitment, exposure, follow-up, and data collection	Methods: Study Design and Setting
Participants	6	(*a*) Give the eligibility criteria, and the sources and methods of selection of participants	Methods: Participants, Eligibility Criteria, and Sampling
Variables	7	Clearly define all outcomes, exposures, predictors, potential confounders, and effect modifiers. Give diagnostic criteria, if applicable	Methods: Variables, Data Sources, and Measurement
Data sources/ measurement	8*	For each variable of interest, give sources of data and details of methods of assessment (measurement). Describe comparability of assessment methods if there is more than one group	Methods: Variables, Data Sources, and Measurement
Bias	9	Describe any efforts to address potential sources of bias	Methods: Bias
Study size	10	Explain how the study size was arrived at	Methods: Participants, Eligibility Criteria, and Sampling
Quantitative variables	11	Explain how quantitative variables were handled in the analyses. If applicable, describe which groupings were chosen and why	Methods: Handling of Variables and Data Cleaning
Statistical methods	12	(*a*) Describe all statistical methods, including those used to control for confounding	Methods: Data Analysis, Missing Data
		(*b*) Describe any methods used to examine subgroups and interactions	
		(*c*) Explain how missing data were addressed	
		(*d*) If applicable, describe analytical methods taking account of sampling strategy	Not applicable
		(<u>*e*</u>) Describe any sensitivity analyses	

**Results**			
Participants	13*	(a) Report numbers of individuals at each stage of study—eg numbers potentially eligible, examined for eligibility, confirmed eligible, included in the study, completing follow-up, and analysed	Results: first paragraph, Figure 1
		(b) Give reasons for non-participation at each stage	
		(c) Consider use of a flow diagram	
Descriptive data	14*	(a) Give characteristics of study participants (eg demographic, clinical, social) and information on exposures and potential confounders	Results: Table 1
		(b) Indicate number of participants with missing data for each variable of interest	Methods: Missing Data, Table S3-6 in Multimedia Appendix 2
Outcome data	15*	Report numbers of outcome events or summary measures	Results: Tables 2–8
Main results	16	(*a*) Give unadjusted estimates and, if applicable, confounder-adjusted estimates and their precision (eg, 95% confidence interval). Make clear which confounders were adjusted for and why they were included	Results: Tables 2–8
		(*b*) Report category boundaries when continuous variables were categorized	Not applicable
		(*c*) If relevant, consider translating estimates of relative risk into absolute risk for a meaningful time period	
Other analyses	17	Report other analyses done—eg analyses of subgroups and interactions, and sensitivity analyses	Results, Multimedia Appendix 2
**Discussion**			
Key results	18	Summarise key results with reference to study objectives	Discussion: first paragraph
Limitations	19	Discuss limitations of the study, taking into account sources of potential bias or imprecision. Discuss both direction and magnitude of any potential bias	Strengths and Limitations
Interpretation	20	Give a cautious overall interpretation of results considering objectives, limitations, multiplicity of analyses, results from similar studies, and other relevant evidence	Discussion
Generalisability	21	Discuss the generalisability (external validity) of the study results	Strengths and Limitations; Discussion
**Other information**			
Funding	22	Give the source of funding and the role of the funders for the present study and, if applicable, for the original study on which the present article is based	Funding and Acknowledgments
