# Supplementary material for: Digital Health Applications for Mental Disorders in Germany Among Users and Nonusers on Actual and Intended Use, Self-Reported Completion, and Reasons for Discontinuation: Cross-Sectional Survey
Source: J Med Internet Res. 2026 Oct 29;28:e99464. doi: 10.2196/99464 (PMC13760326; doi:10.2196/99464)
Supplement: Supplementary file 2 [file jmir_v28i1e99464_app2.docx]

Multimedia Appendix 2: Methods and subgroup analyses

Methods

***Table S1: Overview, definitions, and measurement levels of subgroup variables used in subgroup analyses in a cross-sectional survey on DiGA for mental disorders.***

| **Subgroup** | **Levels** | **Definition** | **Measurement scale** |
| --- | --- | --- | --- |
| **Gender*** | 0 = male 1 = female | 0 = A1 1 = A2 | nominal (2 categories) |
| **Age group** | 0 = 18-40 years 1 = 41-60 years 2 = >=61 years | 0 = A1/2/3 1 = A4/5 2 = A6/7 | ordinal  (>2 categories) |
| **Place of residence** | 0 = rural (up to 20,000 inhabitants) 1 = medium-sized city (20,000 to 100,000 inhabitants) 2 = urban (100,000 or more inhabitants) | 0 = A1/2 1 = A3 2 = A4/5 | nominal (>2 categories) |
| **Education level** | 0 = no completed vocational training or academic degree 1 = company-based vocational training and further training 2 = university / university of applied sciences degree | 0 = A1/2  1 = A3/4/5  2 = A6/7 | nominal (>2 categories) |
| *Gender-based subgroup analyses were restricted to participants who reported male or female gender because the number of participants identifying as diverse was too small for separate subgroup analyses. | | | |

**Table S2: Statistical tests and measures of association used for exploratory subgroup analyses according to the measurement levels of questionnaire variables and subgroup variables.**

|  | **Level of measurement of the subgroup variable** | | |
| --- | --- | --- | --- |
| **Level of measurement of the question** | **Nominal**  (2 categories) | **Nominal**  (>2 categories) | **Ordinal** |
| **Nominal**  (2 categories) | Chi-square test; Phi coefficient | Chi-square test;  Cramer's V | Chi-square test;  Cramer's V |
| **Nominal**  (>2 categories) | Chi-square test;  Cramer's V | Chi-square test;  Cramer's V | Chi-square test;  Cramer's V |
| **Ordinal** | Mann-Whitney-U test; Rank-biserial correlation | Kruskal–Wallis test; Kendall's tau-c | Kruskal–Wallis test; Kendall's tau-c |

Missingness

**Table S3: Results of Little’s Missing Completely at Random Tests for Prespecified Variable Blocks Among Users and Nonusers in a Cross-Sectional Survey DiGA for Mental Disorders**

| **Study group and**  **variable block** | **Included variables** | **χ²** | **df** | **P-value*** | **Missingness patterns** | |
| --- | --- | --- | --- | --- | --- | --- |
| **Users** |  |  |  |  |  |  |
| **Sociodemographic variables** | Gender, age, place of residence, level of education | 12.6 | 14 | .555 | 7 |  |
| **General questionnaire variables** | Completion status, usage duration, weekly time, usage locations | 38.8 | 31 | .159 | 9 |  |
| **Experienced reasons**  **for discontinuation** | Ten experienced discontinuation items | 467 | 380 | <.001 | 63 |  |
| **Non-users** |  |  |  |  |  |  |
| **Sociodemographic variables** | Gender, age, place of residence, level of education | 12.6 | 18 | .816 | 10 |  |
| **General questionnaire variables** | DiGA as an option, weekly time, possible usage locations | 94.8 | 17 | <.001 | 7 |  |
| **Hypothetical reasons**  **for discontinuation**** | Ten hypothetical discontinuation items | 258 | 241 | .214 | 43 |  |
| *A statistically significant Little’s MCAR test provides evidence against the assumption that the data were missing completely at random. A nonsignificant result indicates that the MCAR hypothesis could not be rejected.  **For the analysis of experienced reasons for discontinuation, only users who reported not having completed the DiGA or who were unsure about completion were included. | | | | | |  |

**Table S4: Valid and missing responses for central questionnaire items among users and non-users in a cross-sectional survey on DiGA for mental disorders.**

| **Variable** | **Users** | | | **Non-users** | | |
| --- | --- | --- | --- | --- | --- | --- |
|  | **eligible n** | **valid n** | **missing n (%)** | **eligible n** | **valid n** | **missing n (%)** |
| **Gender** | 769 | 678 | 87 (11.8) | 644 | 571 | 70 (11.3) |
| **Age group** | 769 | 682 | 87 (11.3) | 644 | 579 | 65 (10.1) |
| **Place of residence** | 769 | 682 | 87 (11.3) | 644 | 576 | 68 (10.6) |
| **Education** | 769 | 654 | 115 (15.0) | 644 | 529 | 115 (17.9) |
| **Note.** Eligible n refers to the number of included respondents for whom the respective question was part of the questionnaire. Missing responses include absent or invalid responses after data cleaning. Percentages refer to the eligible sample within each study group. | | | | | | |

**Table S5: Valid and missing responses for usage-related questionnaire variables among users and non-users in a cross-sectional survey on DiGA for mental disorders.**

| **Variable** | **Users** | | | **Non-users** | | |
| --- | --- | --- | --- | --- | --- | --- |
|  | **eligible n** | **valid n** | **missing n (%)** | **eligible n** | **valid n** | **missing n (%)** |
| **DiGA use as an option** | - | - | - | 644 | 589 | 55 (8.5) |
| **Actual and intended weekly time spent** | 769 | 693 | 76 (9.9) | 644 | 553 | 91 (14.1) |
| **Actual and intended locations for use** | 769 | 704 | 65 (8.5) | 644 | 562 | 82 (12.7) |
| **Duration of Use** | 769 | 687 | 82 (10.7) | - | - | - |
| **Completion Status** | 769 | 700 | 69 (9.0) | - | - | - |
|  | | | | | | |

**Table S6: Valid and missing responses for experienced reasons for discontinuation among users and hypothetical reasons for discontinuation among non-users in a cross-sectional survey on DiGA for mental disorders.**

| **Variable** | **Users** | | | **Non-users** | | |
| --- | --- | --- | --- | --- | --- | --- |
|  | **eligible n** | **valid n** | **missing n (%)** | **eligible n** | **valid n** | **missing n (%)** |
| **Desired effect already archived** | 291 | 261 | 30 (10.3) | 644 | 528 | 116 (18.0) |
| **Perceived lack of effectiveness** | 291 | 259 | 32 (11) | 644 | 545 | 99 (15.4) |
| **Incompatibility with daily routines** | 291 | 258 | 33 (11.3) | 644 | 545 | 99 (15.4) |
| **Technical difficulties** | 291 | 267 | 24 (8.2) | 644 | 545 | 99 (15.4) |
| **Boredom during use** | 291 | 263 | 28 (9.6) | 644 | 535 | 109 (16.9) |
| **Insufficient personalization** | 291 | 255 | 36 (12.4) | 644 | 536 | 108 (16.8) |
| **Acute phase of the disorder** | 291 | 259 | 32 (11.0) | 644 | 535 | 109 (16.9) |
| **Lack of motivation** | 291 | 272 | 19 (6.5) | 644 | 544 | 100 (15.5) |
| **Mismatch with individual condition** | 291 | 242 | 49 (16.8) | 644 | 540 | 104 (16.1) |
| **General unsuitability of DiGA** | 291 | 244 | 47 (16.2) | 644 | 542 | 102 (15.8) |

For users, eligible n refers to respondents who reported not having completed the DiGA or were unsure about completion status.

Question 1

Would using a DiGA be an option for you?

A) Yes
B) No
C) Unsure

***Table S7: Response distribution for DiGA use as an option among non-users by age group in a cross-sectional survey on DiGA for mental disorders.***

|  | **DiGA use is an option** | | |
| --- | --- | --- | --- |
| **Age group** | **No** | **Yes** | **Unsure** |
| **18-40 years** | 3 (3.0) | 59 (59.6) | 37 (37.4) |
| **41-60 years** | 24 (10.9) | 103 (46.8) | 93 (42.3) |
| **>=61 years** | 40 (16.3) | 88 (35.8) | 118 (48.0) |
| **Total** | 67 (11.9) | 250 (44.2) | 248 (43.9) |
| N=565 | | | |

***Table S8: Results of exploratory subgroup analyses of DiGA use as an option among non-users in a cross-sectional survey on DiGA for mental disorders.***

|  | **N** | **Chi-square** | **P-value** | **Phi** | **Cramers-V** |
| --- | --- | --- | --- | --- | --- |
| **Gender** | 556 | 2.186 | .335 | 0.063 | - |
| **Age Group** | 565 | 22.306 | <.001 | - | 0.140 |
| **Place of residence** | 562 | 5.389 | .250 | - | 0.069 |
| **Education** | 517 | 2.800 | .592 | - | 0.052 |
| N = 589 Note: “Unsure” responses were excluded from the exploratory subgroup analyses. | | | | | |

Question 2

User: How many minutes per week did you use the DiGA?

Non-user: How many minutes per week would you be willing to use a DiGA?

1) Less than 30 minutes
2) 30-60 minutes
3) 60-90 minutes
4) More than 90 minutes
5) Unsure

Question 3

User: Where did you use the DiGA?

Non-User: Where would you use a DiGA?
Multiple responses were permitted.

1. At home
2. While commuting
3. At work

Question 4

User: For how long did you use the DiGA?

1) Less than 1 week
2) 1-4 weeks
3) 5-8 weeks
4) 9-12 weeks
5) Longer than 12 weeks
6) Unsure

**Table S9: Response distribution for reported duration of active DiGA use among users by age group in a cross-sectional survey on DiGA for mental disorders.**

|  | **Duration of active DiGA in weeks** | | | | | | |
| --- | --- | --- | --- | --- | --- | --- | --- |
| **Age group** | **Less than 1** | | **1-4** | **5-8** | **9-12** | **More than 12** | **Unsure** |
| **18-40 years** | 6 (2.4) | 67 (27.3) | | 65 (26.5) | 53 (21.6) | 37 (15.1) | 17 (6.9) |
| **41-60 years** | 3 (1.0) | 41 (13.6) | | 60 (19.9) | 98 (32.5) | 75 (24.8) | 25 (8.3) |
| **≥61 years** | 1 (0.9) | 12 (10.9) | | 23 (20.9) | 41 (37.3) | 20 (18.2) | 13 (11.8) |
| **Total** | 10 (1.5) | 120 (18.3) | | 148 (22.5) | 192 (29.2) | 132 (20.1) | 55 (8.4) |
| N=657 | | | | | | | |

***Table S10: Statistical results of exploratory subgroup analyses of reported duration of active DiGA use among users in a cross-sectional survey on DiGA for mental disorders.***

| **Subgroup** | **n** | **Chi-square** | **P-value** | **Cramer's V** |
| --- | --- | --- | --- | --- |
| **Gender** | 653 | 0.753 | .980 | 0.034 |
| **Age group** | 657 | 40.113 | <.001 | 0.175 |
| **Place of residence** | 658 | 14.670 | .145 | 0.106 |
| **Education level** | 614 | 13.092 | .219 | 0.103 |
| N = 687 | | | | |

Question 5

User: Did you complete your DiGA program?

A) Yes
B) No
C) Unsure

***Table S11: Response distribution for self-reported completion of DiGA use among users by age group in a cross-sectional survey on DiGA for mental disorders.***

|  | **self-reported completion of DiGA program** | | |
| --- | --- | --- | --- |
| **Age group** | **No** | **Yes** | **Unsure** |
| **18-40 years** | 126 (50.4) | 111 (44.4) | 13 (5.2) |
| **41-60 years** | 90 (29.4) | 203 (66.3) | 13 (4.2) |
| **≥61 years** | 22 (19.6) | 85 (75.9) | 5 (4.5) |
| **Total** | 238 (35.6) | 399 (59.7) | 31 (4.6) |

N=668

***Table S12: Results of exploratory subgroup analyses of self-reported completion of DiGA use among users in a cross-sectional survey on DiGA for mental disorders.***

| **Subgroup** | **N** | **Chi-square** | **P-Value** | **Phi** | **Cramer's V** |
| --- | --- | --- | --- | --- | --- |
| **Gender** | 664 | 0.254 | .881 | 0.02 | - |
| **Age group** | 668 | 43.913 | <.01 | - | 0.181 |
| **Place of residence** | 668 | 7.076 | .132 | - | 0.073 |
| **Education level** | 626 | 5.392 | .249 | - | 0.066 |
| N = 700 | | | | | |

**Table S13: Reported duration of active DiGA use by self-reported completion status among users in a cross-sectional survey on DiGA for mental disorders.**

|  | **Duration of active use, weeks** | | | | | | |
| --- | --- | --- | --- | --- | --- | --- | --- |
| **reported completion** | **Less than 1** | **1-4** | **5-8** | **9-12** | **>12** | **Unsure** | **Total** |
| **Yes** | 0 (0.0) | 22 (5.6) | 68 (17.2) | 156 (39.4) | 110 (27.8) | 40 (10.1) | 396 |
| **No** | 12 (4.7) | 104 (40.9) | 75 (29.5) | 34 (13.4) | 15 (5.9) | 14 (5.5) | 254 |
| **Unsure** | 1 (3.4) | 3 (10.3) | 9 (31.0) | 7 (24.1) | 5 (17.2) | 4 (13.8) | 29 |
| **Total** | 13 (1.9) | 129 (19.0) | 152 (22.4) | 197 (29.0) | 130 (19.1) | 58 (8.5) | 679 |

N=679

Question 6

User: I did not continue using the DiGA because…

A) the effect I had desired for had already occurred.
B) no effectiveness was perceived.
C) the DiGA was not compatible with my daily routines.
D) the DiGA had technical difficulties.
E) I got bored during using it.
F) the DiGA could not be sufficiently personalized.
G) I was going through an acute phase of my disorder.
H) I lacked motivation to use it.
I) the DiGA did not match my condition.
J) a DiGA in general is not suitable for me.

***Table S14: Response distribution for lack of motivation as an experienced reason for discontinuation among users by age group in a cross-sectional survey on DiGA for mental disorders.***

|  | I lacked motivation to use it. | | | | |
| --- | --- | --- | --- | --- | --- |
| **Age group** | **Strongly disagree** n (%) | **Rather Disagree** n (%) | **partly applies,**  **partly does not apply** n (%) | **Rather Agree** n (%) | **Strongly Agree** n (%) |
| **18-40 years** | 9.8% (13) | 3.8% (5) | 18.8% (25) | 32.3% (43) | 35.3% (47) |
| **41-60 years** | 15.3% (15) | 9.2% (9) | 32.7% (32) | 27.6% (27) | 15.3% (15) |
| **≥61 years** | 23.8% (5) | 4.8% (1) | 42.9% (9) | 14.3% (3) | 14.3% (3) |
| **Total** | 13.1% (33) | 6.0% (15) | 26.2% (66) | 29% (73) | 25.8% (65) |
| N=289 | | | | | |

***Table S15: Response distribution for boredom during use as an experienced reason for discontinuation among users by age group in a cross-sectional survey on DiGA for mental disorders.***

|  | **I got bored during using it.** | | | | |
| --- | --- | --- | --- | --- | --- |
| **Age group** | **Strongly disagree n (%)** | **Rather Disagree n (%)** | **partly applies,**  **partly does not apply n (%)** | **Rather Agree n (%)** | **Strongly Agree n (%)** |
| **18-40 years** | 25.8% (33) | 9.4% (12) | 22.7% (29) | 25.0% (32) | 17.2% (22) |
| **41-60 years** | 33.0% (32) | 14.4% (14) | 28.9% (28) | 14.4% (14) | 9.3% (9) |
| **≥61 years** | 35.0% (7) | 10.0% (2) | 40.0% (8) | 10.0% (2) | 5.0% (1) |
| Total | 29.4% (72) | 11.4% (28) | 26.5% (65) | 19.6% (48) | 13.1% (32) |
| N=289 | | | | | |

**Table S16: Subgroup analysis by age group for experienced reasons for discontinuation among users in a cross-sectional survey on DiGA for mental disorders.**

| **Variable** | **n** | **Kruskal-Wallis H** | **P-Value** | **Tau-C** |
| --- | --- | --- | --- | --- |
| I got bored during using it. | 245 | 7.583 | .023 | -0.15 |
| I lacked motivation to use it. | 252 | 20.233 | <.001 | -0.25 |

**Table S17: Post hoc subgroup comparisons by age group for experienced reasons for discontinuation among users in a cross-sectional survey on DiGA for mental disorders.**

| **Variable** | **Comparison** | **Z-Value** | **P-Value** |
| --- | --- | --- | --- |
| I got bored during using it. | 0 - 1 | 2.471 | .013 |
|  | 0 - 2 | 1.776 | .076 |
|  | 1 - 2 | 0.385 | .700 |
| I lacked motivation to use it. | 0 - 1 | 3.919 | <.001 |
|  | 0 - 2 | 3.086 | .002 |
|  | 1 - 2 | 0.844 | .399 |
| N = 289; 0 = 18-40, 1=41-60, 2= >=61 | | | |

***Table S18: Response distribution for insufficient personalization as an experienced reason for discontinuation among users by gender in a cross-sectional survey on DiGA for mental disorders.***

|  | **the DiGA could not be sufficiently personalized.** | | | | |
| --- | --- | --- | --- | --- | --- |
| **Gender** | **Strongly disagree n (%)** | **Rather Disagree n (%)** | **partly applies,**  **partly does not apply n (%)** | **Rather Agree n (%)** | **Strongly Agree n (%)** |
| **Male** | 31.9% (23) | 13.9% (10) | 20.8% (15) | 26.4% (19) | 6.9% (5) |
| **Female** | 16.7% (28) | 11.9% (20) | 23.2% (39) | 23.2% (39) | 25.0% (42) |
| **Total** | 21.2% (51) | 12.5% (30) | 22.5% (54) | 24.2% (58) | 19.6% (47) |
| N= 240 | | | | | |

**Table S19: Subgroup analysis by gender for insufficient personalization as an experienced reason for discontinuation among users in a cross-sectional survey on DiGA for mental disorders.**

| **Variable** | **U-Value** | **P-Value** | **r_rb** |
| --- | --- | --- | --- |
| the DiGA could not be sufficiently personalized. | 7623 | <.001 | 0.26 |
| N = 240 | | | |

Question 7

User: I would not continue using a DiGA, if…

A) the effect I had desired had already occurred.
B) no improvements were perceived from using it.
C) it was not compatible with my daily routines.
D) it had technical difficulties.
E) I got bored while using it.
F) it could not be sufficiently personalized.
G) I was going through an acute phase of my disorder.
H) I lacked motivation to use it.
I) it did not match my condition.
J) it is general is not suitable for me.

***Table S20: Response distribution for incompatibility with daily routines as a hypothetical reason for discontinuation among non-users by age group in a cross-sectional survey on DiGA for mental disorders.***

|  | **it was not compatible with my daily routines.** | | | | |
| --- | --- | --- | --- | --- | --- |
| **Age group** | **Strongly disagree** n (%) | **Rather Disagree** n (%) | **partly applies,**  **partly does not apply** n (%) | **Rather Agree** n (%) | **Strongly Agree** n (%) |
| **18-40 years** | 3.2% (3) | 5.3% (5) | 12.6% (12) | 28.4% (27) | 50.5% (48) |
| **41-60 years** | 4.8% (10) | 5.7% (12) | 13.4% (28) | 22.0% (46) | 54.1% (113) |
| **≥61 years** | 8.1% (18) | 8.5% (19) | 21.1% (47) | 23.3% (52) | 39.0% (87) |
| **Total** | 5.9% (31) | 6.8% (36) | 16.5% (87) | 23.7% (125) | 47.1% (248) |
| N= 527 | | | | | |

***Table S21: Response distribution for mismatch with the individual condition as a hypothetical reason for discontinuation among non-users by age group in a cross-sectional survey on DiGA for mental disorders.***

|  | **it did not match my condition.** | | | | |
| --- | --- | --- | --- | --- | --- |
| **Age group** | **Strongly disagree n (%)** | **Rather Disagree n (%)** | **partly applies,**  **partly does not apply n (%)** | **Rather Agree n (%)** | **Strongly Agree n (%)** |
| **18-40 years** | - | 3.2% (3) | 9.6% (9) | 18.1% (17) | 691% (65) |
| **41-60 years** | 4.3% (9) | 2.9% (6) | 9.6% (20) | 16.3% (34) | 67% (140) |
| **≥61 years** | 9.1% (20) | 4.6% (10) | 11.9% (26) | 21.5% (47) | 53.0% (116) |
| **Total** | 5.6% (29) | 3.6% (19) | 10.5% (55) | 18.8% (98) | 61.5% (321) |
| N= 522 | | | | | |

**Table S22: Subgroup analysis by age group for anticipated reasons for discontinuation among non-users in a cross-sectional survey on DiGA for mental disorders.**

| **Variable** | **Kruskal-Wallis H** | **P-Value** | **Tau-C** |
| --- | --- | --- | --- |
| it was not compatible with my daily routines. | 13.939 | <.001 | -0.13 |
| it did not match my condition. | 13.870 | <.001 | -0.13 |
| N = 561 | | | |

**Table S23: Post hoc subgroup comparisons by age group for hypothetical reasons for discontinuation among non-users in a cross-sectional survey on DiGA for mental disorders.**

| **Variable** | **Comparison** | **Z-Value** | **P-Value** |
| --- | --- | --- | --- |
| it was not compatible with my daily routines. | 0 - 1 | -0.097 | .922 |
|  | 0 - 2 | 2.618 | <.01 |
|  | 1 - 2 | 3.457 | <.01 |
| it did not match my condition. | 0 - 1 | 0.618 | .537 |
|  | 0 - 2 | 3.071 | <.01 |
|  | 1 - 2 | 3.122 | <.01 |
| N = 561; 0 = 18-40, 1=41-60, 2= >=61 | | | |
