# Supplementary material for: Digital Health Applications for Mental Disorders in Germany Among Users and Nonusers on Actual and Intended Use, Self-Reported Completion, and Reasons for Discontinuation: Cross-Sectional Survey
Source: J Med Internet Res. 2026 Oct 29;28:e99464. doi: 10.2196/99464 (PMC13760326; doi:10.2196/99464)
Supplement: Supplementary file 3 [file jmir_v28i1e99464_app3.docx]

Multimedia Appendix 2: Free-Text Responses on Additional Reasons for Discontinuation

Participants in both study groups could provide additional reasons for discontinuation in optional free-text fields. The following tables reproduce all nonempty responses. The responses were not coded, categorized, or included in the quantitative or qualitative analyses. Potentially identifying information was removed. Responses were originally provided in German and are presented together with an English translation.

**Table S1: Additional Experienced Reasons for Discontinuation Reported by Users**

| **Response Number** | **Original German Response** | **English translation** |
| --- | --- | --- |
| User 1 | "Die Lektion ""Exposition mit meinen Ängsten"" hat mich stark beeinträchtigt/geängstigt und eine längere Vermeidungsphase verursacht.  Hier war es sehr wichtig für mich, zeitgleich an meiner regelmäßigen begleitenden Psychotherapie teilzunehmen." | "The lesson ‘Exposure to My Fears’ had a significant negative impact on me and caused me to feel anxious, leading to a prolonged period of avoidance.  It was very important for me to continue attending my regular psychotherapy sessions at the same time." |
| User 2 | Es gab technische Probleme. Ich konnte die App nicht mehr benutzen. | There were technical issues. I couldn't use the app anymore. |
| User 3 | Wusste nicht, dass ich es nur 90 Tage nutzen kann, deswegen nicht zu Ende geschafft | I didn't know I could only use it for 90 days, so I didn't finish it |
| User 4 | die DiGA war für Menschen, die noch keine Erfahrungen im Umgang mit einer Psychotherapie hatten. Ich hatte die bereits und so wurde es schnell langweilig | The DiGA was for people who had no prior experience with psychotherapy. I already had that experience, so it quickly became boring. |
| User 5 | Siehe Einträge vorher: katastrophale Nutzererfahrung, weil Oberflächen nicht gingen, Rechtschreibfehler in den Texten waren was super unprofessionell ist, die Texte null zur Problematik gepasst haben und in die Opferstellung gingen und bei der Nutzung negative Gefühle ausgelöst haben. Aber ein perfektes Beispiel wie eine DiGA nicht sein sollte. Es braucht positive Erfahrung und keine Babysprache mit erwachsenen Menschen. | See previous entries: a disastrous user experience because the interfaces didn’t work, there were spelling errors in the text—which is extremely unprofessional—the text had nothing to do with the issue at hand, adopted a victim mentality, and triggered negative feelings during use. But it’s a perfect example of how a digital health application should not be. It needs to provide a positive experience, not use baby talk with adults. |
| User 6 | Das Programm war zeitlich nicht mit meiner spezifischen Situation vereinbar. Dass man durch besondere Lebensumstände nur einen begrenzten Zeitraum zur Verfügung hat, den man intensiv nutzen möchte, war in der App nicht vorgesehen. Die gesperrten Einheiten mit ihren Wartezeiten sind nett in der Theorie, aber waren für mich nicht sinnvoll. Somit konnte ich das Programm leider nur zu einem Viertel machen. | The program’s schedule wasn’t compatible with my specific situation. The app didn’t account for the fact that, due to special circumstances, I only had a limited amount of time available that I wanted to use intensively. The locked sessions with their waiting periods are nice in theory, but they didn’t make sense for me. As a result, I was unfortunately only able to complete a quarter of the program. |
| User 7 | Ich habe den letzten Tag versäumt. Vielleicht hätte ich eine Erinnerungsmail gebraucht. | I missed the last day. Maybe I could have used a reminder email. |
| User 8 | Teilweise die Verbindung zur DIGA abgebrochen wurde und dann nicht mehr verfügbar war. | At times, the connection to DIGA was interrupted and was then no longer available. |
| User 9 | Der Zeitraum war abgelaufen, ich hätte sie sonst gerne weiterverwendet. Ich hätte mir auch gerne manche Sachen ausgedruckt, ich habe aber keinen Drucker. Manchmal wäre es besser gewesen, wenn ich die Anwendung am PC statt am Handy nutzen könnte. | The subscription period had expired; otherwise, I would have liked to keep using it. I also would have liked to print out some things, but I don't have a printer. Sometimes it would have been better if I could have used the app on my computer instead of my phone. |
| User 10 | Man kann in der App Somnio immer nur nach bestimmter Zeit das nächste Stück erlernen, währe d dieser Zeit setzt man sich trotzdem schon mit den Schlafstörungen auseinander. Ich bin trotz Verlängerung nicht ganz bis zum Schluss gekommen, profitiere aber immer noch vin dem Erlernten | In the Somnio app, you can only move on to the next lesson after a certain amount of time has passed, but during that time, you’re still working through your sleep issues. Even though I extended the program, I didn’t quite make it all the way to the end, but I’m still benefiting from what I’ve learned. |
| User 11 | Ich habe mehr Zeit gebraucht | I would have needed more time. |
| User 12 | "Mir fehlte manchmal eine richtige Interaktion miteinander.  Ich hätte gerne manchmal Fragen stellen wollen zwischendurch.  Ich wusste nicht, dass es zeitlich eingeschränkt ist die app zu nutzen." | "Sometimes I felt like we weren't really interacting with each other.  There were times when I would have liked to ask questions here and there. I didn't know there was a time limit on using the app." |
| User 13 | Ich hätte eine Erinnerungsfunktion benötigt und vorab einen Plan, wie genau der Ablauf ist, wie viele „Einheiten“, welche Themen. Ich habe zumindest nicht wahrgenommen, dass es das gab. | I would have needed a reminder feature and a plan laid out in advance detailing exactly how the process works, how many “sessions,” and what topics would be covered. At least, I didn’t notice that any of that was available. |
| User 14 | Absolut keine Perspektive auf eine Hilfestellung. Eine tägliche Befragung aus der ich ein 1-10 Ranking bekomme wie es mir heute geht? Wie hängen geblieben muss man sein zu erwarten dass das irgendeine Form der Veränderung erwirkt. War nach zweimaliger Nutzung zu sauer um die App nicht zu deinstallieren. Alles daran war schlimm, vor allem weil die Hoffnung auf Abhilfe derart herb enttäuscht wurde. | Absolutely no prospect of getting any help. A daily survey where I’m given a 1–10 rating of how I’m doing today? How stuck do you have to be to expect that to bring about any kind of change? After using it twice, I was too frustrated not to uninstall the app. Everything about it was terrible, especially because my hope for relief was so bitterly dashed. |
| User 15 | Ich habe bereits viel Therapieerfahrung, daher half mir die App zwar, einige Aspekte der Psychoedukation aufzufrischen, aber mir fehlte die Interaktion mit und Anleitung durch eine:n Therapeut:in. Es fiel mir sehr schwer, mich alleine zu Übungen zu motivieren. | I already have a lot of experience with therapy, so while the app helped me brush up on some aspects of psychoeducation, I missed the interaction with and guidance from a therapist. I found it very difficult to motivate myself to do the exercises on my own. |
| User 16 | Bin Vater geworden und da war kein Schlaf mehr planbar bzw. sehr unregelmäßig. Bis dahin war die App ganz gut. | I became a father, and after that, I couldn't plan my sleep anymore—or rather, it became very irregular. Up until then, the app had been pretty good. |
| User 17 | Die DiGA hat mit mir kommuniziert, als wäre ich stark kognitiv eingeschränkt. Außerdem war der simple ChatBot dahinter weit weniger komplex, als vom Anbieter behauptet und es wirkte recht stumpf und stupide. | DiGA communicated with me as if I had severe cognitive impairments. Furthermore, the simple chatbot behind it was far less complex than the provider claimed, and it came across as rather dull and mindless. |
| User 18 | Nicht unterhaltsam genug. Viel selbst zu lesen... Bei Depression mit Stresssymptomen zu Zeitaufwendig... Man findet dennoch keine Ruhe. | Not entertaining enough. Too much to read on your own... Too time-consuming if you're dealing with depression and stress symptoms... You still can't find any peace. |
| User 19 | "Technische Mängel, Anmeldung ging verloren  Zu viel Text zum Lesen, zu wenig Bilder und Videos zur Erklärung.  Lesen ist an manchen Tagen für Depressive oft zu anstrengend." | "Technical issues, registration was lost  Too much text to read, not enough pictures and videos to explain things.  On some days, reading is often too exhausting for people with depression." |
| User 20 | Bei einer akuten Depression eine App zu entwickeln in der man Entscheidungen mit Ausschlussverfahren treffen muss, die aber angeblich speziell für Depressionen ist. Die App ist altbacken, unsympathisch und unseriös. | To develop an app for acute depression that requires users to make decisions through a process of elimination, even though it's supposedly designed specifically for depression. The app is outdated, unappealing, and unprofessional. |
| User 21 | konnte leider meinen Account nicht in die zweite Bewilligung mitnehmen, da mir die Information über den Verlust der Daten bei Ablauf der Nutzungsdauer zu spät bekannt war. darüber sollte deutlicher hingewiesen werden | Unfortunately, I was unable to transfer my account to the second authorization period because I found out too late that my data would be lost once the usage period expired. This should be made clearer. |
| User 22 | Ich hatte schon früher über die Krankheit viel gelesen, ich bin seit mehreren Jahren im Thema. Es war also nichts neues für mich. Kann mir aber vorstellen, dass für diejenigen, die noch keine Erfahrung mit ihrer Krankheit haben, eine DiGA wirklich viel bringt. | I had already read a lot about the disease before; I've been following the topic for several years. So it wasn't anything new to me. But I can imagine that for those who haven't yet had any experience with their illness, a digital health application (DiGA) can really be a big help. |
| User 23 | Generiert antworten der KI sind für mich nur Geschwafel. | To me, AI-generated responses are just a bunch of nonsense. |
| User 24 | Begrenzter Zugangszeitraum | Limited Access Period |
| User 25 | Wie gesagt, wie Deprexis waren Texte viel zu lang und zu kompliziert. Und es hat mich getriggert.  Die rücken app habe ich bis zuende genutzt | Like I said, just like Deprexis, the texts were way too long and too complicated. And it set me off.  I used the Rücken app until the end. |
| User 26 | ersetzt leider keine Persönliche Therapie, zur Überbrückung ok, da akute Phase: stationäre Therapie notwendig | Unfortunately, it is no substitute for in-person therapy; it's okay as a temporary measure, since this is the acute phase: inpatient therapy is necessary |
| User 27 | War nicht das Richtige für mich. Nur am PC nutzbar. Für mich nicht sehr reizvoll gestaltet | It wasn't right for me. It can only be used on a computer. The design isn't very appealing to me. |
| User 28 | Zeit Ablauf der Verordnung | Expiration Date of the Prescription |
| User 29 | Zeitrahmen in der die Anwendung nutzbar war war abgelaufen, aber Krankheit so verbessert durch Medikation, dass das nicht abschließen kein Problem war. | The time frame during which the app was available had expired, but my condition had improved so much thanks to medication that not completing it wasn't a problem. |
| User 30 | Wenn man unter einer Depression, bei mir zusätzlich begleitet von einem ADS, ist es schwierig bei solchen Anwendungen am Ball zu bleiben. Für mich war eigentlich relativ schnell klar, dass ich es sowieso nicht durchhalten werde. Leider habe ich immer gedacht "Das weiß ich doch eh schon und es bringt nichts", wenn ich Inhalte bearbeitet habe und mich denen nicht angenommen. Besonders für Menschen mit ähnlichen Erkrankungen, wird es immer schwierig sein, solche Dinge eigenständig anzugehen. | When you’re dealing with depression—which, in my case, is compounded by ADHD—it’s hard to stay on track with these kinds of programs. It actually became clear to me relatively quickly that I wouldn’t be able to stick with it anyway. Unfortunately, whenever I worked through the material, I’d always think, “I already know this, and it’s not helping,” and I wouldn’t really engage with it. It will always be difficult, especially for people with similar conditions, to tackle these kinds of things on their own. |
| User 31 | Ist eher was für Personen, die auf einen Psychotherapieplatz warten und etwas machen können und wollen. Für mich waren die enthaltenen Infos und Übungen „ein alter hut“, da ich schon länger in Therapie war und die Apps nur aus Neugier probiert habe. Ein paar interessante Sachen waren für mich dabei. Grundlegende Probleme bespricht man aber lieber mit Menschen und nicht mit Apps. | It’s more for people who are waiting for a psychotherapy spot and are able and willing to do something. For me, the information and exercises included were “old hat,” since I’d already been in therapy for quite some time and only tried the apps out of curiosity. There were a few interesting things in there for me. But it’s better to discuss fundamental problems with people rather than with apps. |
| User 32 | Der Zeitraum der Nutzung war abgelaufen. | The usage period had expired. |
| User 33 | Mir war der "Ansprechpartner" unsympathisch. Es ist mir unangenehm über mein Befinden mit einem Computer zu kommunizieren. | I didn't like the “contact person.” I find it uncomfortable to communicate with a computer about how I'm feeling. |
| User 34 | Motivation fehlte. Anders als in der Psychotherapie hatte ich keine festen Termine, wodurch ich die Nutzung schleifen liess. | I lacked motivation. Unlike in psychotherapy, I didn't have set appointments, so I let my use of the app slide. |
| User 35 | Freischaltcode war abgelaufen. | The activation code had expired. |
| User 36 | Ich habe die Tage erst nach dem 90. Tag verlängern können. Das Programm erkannte mich nicht, so dass ich wieder von vorn beginnen musste und die anschl. Benefits nicht nutzen kann. Außerdem wurde es langweilig, dasselbe Programm 2x zu durchlaufen, das zerstört die Illusion der persönlichen Ansprache. Entsprechend war der zweite Durchlauf wenig ergiebig. Ich würde gerne dauerhaft mit der DiGA arbeiten. | I wasn't able to extend the program until after the 90th day. The program didn't recognize me, so I had to start over from the beginning and couldn't take advantage of the subsequent benefits. Plus, it got boring going through the same program twice—it ruins the illusion of a personalized experience. As a result, the second round wasn't very productive. I'd like to continue using the DiGA on a long-term basis. |
| User 37 | Meine Angststörung hat sich rund um das Thema Sterben. Tod, "nicht mehr sein" gedreht. Hier gab es wenig Anwendung für. Für andere Ängste sicherlich super. | My anxiety disorder revolved around the topics of dying, death, and “no longer being.” This approach wasn’t very helpful for that. It’s certainly great for other fears. |
| User 38 | Zu langatmig, zu steif (unflexibel). | Too long-winded, too stiff (inflexible). |
| User 39 | Ich habe einen Platz in der Tagesklinik bekommen und wollte mich dann voll darauf konzentrieren. | I got a spot at the day clinic and wanted to focus all my attention on that. |
| User 40 | Ich habe die App begleitend zur Therapie genutzt und das wurde mir leider etwas zu viel! Ohne Therapie hätte ich die App dauerhaft gemacht. | I used the app as part of my therapy, and unfortunately, it became a bit too much for me! If it hadn't been for the therapy, I would have kept using the app long-term. |
| User 41 | Deprexis schien mir ungeeignet, in meiner Wahrnehmung oberflächlich, allg. bekannte Infos, teilweise fast kindisch. | Deprexis seemed unsuitable to me—in my view, it was superficial, full of common knowledge, and at times almost childish. |
| User 42 | Unpassende Aussagen wie "einfach mal an etwas anderes denken" oder "die eigen3e Einstellung wechseln" sind nicht das was mir bei einer Depression/depressiven Phase hilft. Wenn ich das "einfach so" könnte, bräuchte ich die App nicht. :) | Inappropriate statements like “just try thinking about something else” or “change your own attitude” aren’t what helps me when I’m dealing with depression or a depressive episode. If I could do that “just like that,” I wouldn’t need the app. :) |
| User 43 | Priovi hat keine App, sondern nur eine Browserseite. Das macht es mir schwer, an die regelmäßige Nutzung zu denken. | Priovi doesn't have an app; it's just a website. That makes it hard for me to think about using it according to the rules. |
| User 44 | Ich hätte mir ein "Streak-System", wie z.B. bei Snapchat oder Duolingo gewünscht. Da ich keinen Effekt gespürt habe war ich nicht mehr motiviert die DiGA zu benutzen. Mit mehr "Motivationsstupsern" seitens der App wie z.B. Streaks wäre ich wahrscheinlich eher am Ball geblieben. | I would have liked to see a “streak system,” like the ones on Snapchat or Duolingo. Since I didn't feel like it was making a difference, I lost my motivation to use the DiGA. If the app had provided more “motivational nudges”—like streaks—I probably would have stuck with it longer. |
| User 45 | Zu viel Gerede, zu viele "dumme" Optionen. Letzteres führte, egal welche Antwort zum gleichen Ergebnis. | Too much talk, too many “stupid” options. The latter led to the same result no matter which answer you chose. |
| User 46 | War völlig unbrauchbar! Hat die Symptome verschlimmert! Passte nicht zur Diagnose! | It was completely useless! It made the symptoms worse! It didn't match the diagnosis! |
| User 47 | - viel Text für Menschen mit Konzentrationsschwierigkeiten, - Anklickbeispiele passen nicht zur Schwere der Erkrankung, demotivieren eher | Too much text for people who have trouble concentrating, - The click-through examples aren't appropriate for the severity of the condition; they tend to be demotivating |
| User 48 | schwere depressive Episode, dadurch das 12 Wochen genehmigt werden -> Druck hoch -> wäre gut in Krankenphasen pausieren zu können | Severe depressive episode, resulting in a 12-week leave of absence being approved -> High stress -> It would be good to be able to take a break during periods of illness |
| User 49 | War einfach nur nervend! | It was just so annoying! |
| User 50 | Die Anwendungszeit war zu kurz und ich hatte nicht die Motivation jeden Tag in der App zu lesen. | I didn't use the app long enough, and I didn't have the motivation to read in it every day. |
| User 51 | Die Laufzeit war zu kurz. | The duration was too short. |
| User 52 | Bei meiner psychischen Erkrankung hat mir die soziale Interaktion gefehlt. Zumal die DiGA auch nur eine Übergangslösung war, da der Therapeut kein Platz für hatte | Because of my mental illness, I missed social interaction. Especially since the DiGA was only a temporary solution, since the therapist didn't have room for it |
| User 53 | Mir fehlte persönliche Ansprache | I missed the personal touch |
| User 54 | Lizenz vorher abgelaufen, nicht genug Zeit für die Bearbeitung. | My license expired earlier, and I didn't have enough time to process it. I got a spot in therapy and stopped using the app. |
| User 55 | Ich habe einen Therapieplatz bekommen und die App nicht mehr benutzt. | I got a spot in therapy and stopped using the app. |
| User 56 | Es war sehr schwer die DiGA bis zum Ende durchzuführen. Es hat sehr viel Selbstüberwindung gekostet. Ich musste das Rezept verlängern, da ich nach 3 Monaten nicht fertig war. | It was very difficult to complete the DiGA. It took a lot of willpower. I had to extend the prescription because I wasn't finished after 3 months. |
| User 57 | In einem Depressionstief fiel es mir sehr schwer mich alleine mit der App zu beschäftigen und die Aufgaben auszuüben. Die App hatte auch Programmierfehler und ist oft abgestürzt. | When I was in the depths of depression, I found it very difficult to use the app on my own and complete the tasks. The app also had programming errors and crashed frequently. |
| User 58 | Ich bin in eine Psychosomatische Reha gegangen | I went to a psychosomatic rehabilitation center |
| User 59 | Ich habe DiGA genutzt, bis die Therapie bei einer Psychotherapeutin anfing, damit der Input der App nicht die Therapie durcheinanderbringt. (Ich war froh, die App hat mich gelangweilt.) | I used DiGA until I started therapy with a psychotherapist, so that the app's input wouldn't interfere with the therapy. (I was glad—the app bored me.) |
| User 60 | Ich hätte etwas mehr Zeit benötigt, da ich neben dem Berufsleben nicht mehr jeden Tag bzw. regelmäßig damit arbeiten konnte. Außerdem waren die Einheiten in der Anwendung so konzipiert, dass zwischen den Kapiteln einige Tage Pause eingeplant. | I used DiGA until I started therapy with a psychotherapist, so that the app's input wouldn't interfere with the therapy. (I was glad—the app bored me.) |
| User 61 | In Vergessenheit geraten. | Fallen into oblivion. |
| User 62 | Sehr gründliche Aufklärung über Gehirn Funktionen oder wieso und warum Reaktionen so verlaufen aber nicht anders. | A very thorough explanation of brain functions—or why reactions happen the way they do and not differently. |
| User 63 | Meine akute Symptomatik wurde durch eine organische Ursache ausgelöst/hatte keine rein psychischen Grundlagen. Dies war zum Zeitpunkt der DiGA-Verschreibung noch nicht klar -> daher hat mir die Nutzung der DiGA leider nicht viel gebracht. | My acute symptoms were triggered by an organic cause and were not purely psychological in nature. This was not yet clear at the time the DiGA was prescribed -> therefore, unfortunately, using the DiGA did not help me much. |
| User 64 | In der von mir genutzten DiGA mussten durchgehend Fragen in Freitextfeldern beantwortet werden. Dies ist sehr mühselig, insbesondere, wenn man an Depressionen erkrankt ist. Ich habe nicht verstanden, was mit diesen Antworten geschieht. Werden sie von einem/einer Therapeutin gelesen oder dient es nur meiner Reflexion? Was ist der Nutzen, wenn ich keine Reaktion auf meine Antwort erhalte? | In the digital health app I used, I had to answer questions in free-text fields throughout the entire process. This is very tedious, especially if you’re suffering from depression. I didn’t understand what happens to these answers. Are they read by a therapist, or are they just for my own reflection? What’s the point if I don’t get any feedback on my answers? |
| User 65 | Nach der Arbeit zu müde, bei Selbstständigkeit hat man kaum Zeit für private oder gesundheitlich fördernde Angelegenheiten. Zu kurzer Zeitraum 90 Tage ist zu kurz für mich, brauche 3x so viel. | Too tired after work; when you're self-employed, you hardly have any time for personal matters or things that promote good health. The 90-day period is too short for me—I need three times as long. |
| User 66 | Es ergaben sich zu viele Rückfragen, Verständnisprobleme mit der Anwendung der Brille wurde mir übel, bekam dadurch noch mehr Ängste und Unsicherheit. | There were too many questions, and I got fed up with the difficulties in understanding how to use the glasses, which made me even more anxious and insecure. |
| User 67 | Es war in der DiGA vorgesehen, jede Aktivität festzuhalten. Das ist in keinem Leben umsetzbar. Außerdem gab es beim Zeitlimit für dieses Tagebuch was nicht gerade zur Motivation beiträgt. | The DiGA was designed to track every activity. That’s impossible to do in real life. Plus, there was a time limit for this journal, which didn’t exactly help with motivation. |
| User 68 | Für die relativ lange Nutzungsdauer von priovi finde ich umgeleitete Gespräche, bei 2 bis 3 mal pro Woche Nutzung (30-60 Minuten), sehr kurz. Nach 3-4 Monaten ist man durch. Die restlichen 9 Monate kann man nur Übungen wiederholen. Vielleicht könnte man das Gespräch noch etwas ausführlicher gestalten. | Given how long priovi is designed to be used, I find the unguided conversations—with 2 to 3 sessions per week (30–60 minutes each)—to be very short. You’re done after 3–4 months. For the remaining 9 months, you can only repeat the exercises. Perhaps the conversations could be made a bit more detailed. |
| User 69 | Das persönliche Feedback hat mir gefehlt. Manche Antworten passten gar nicht. | I missed the personal feedback. Some of the answers didn't make any sense at all. |

***Table S2: Additional Experienced Reasons for Discontinuation Reported by Non-Users***

| Response Number | Original German Response | English translation |
| --- | --- | --- |
| Non-User 1 | Benutzerunfreundliche UI und Bugs wären eindeutig ein Zeichen der Inkompetenz in der Technik und Medien welches in Deutschland eh eher gängig ist und würde es dann auch nicht nutzten | A user-unfriendly UI and bugs would clearly be a sign of incompetence in technology and media—which is fairly common in Germany anyway—and I wouldn’t use it in that case. |
| Non-User 2 | Im psychotherapeutischen Rahmen finde ich es sehr wichtig, dass die DiGA so individuell wie möglich angepasst wird. Es wäre sogar hilfreich, mit einem menschlichen Ansprechpartner interagieren zu können. Ohne eine personalisierte Anpassung, verliert man die Motivation für eine solche Anwendung schnell. | In a psychotherapeutic setting, I think it’s very important that the DiGA is as customizable as much as possible. It would even be helpful to be able to interact with a human contact person. Without personalized customization, you quickly lose motivation to use such an app. |
| Non-User 3 | Sie müsste auch auf Reisen umsetzbar sein | It would also have to be usable while traveling. |
| Non-User 4 | Tracking meiner Gesundheit für dritte einsehbar, zu hohe Kosten, um eine Verordnung zu erhalten, müsste ich zum Arzt | My health data being visible to third parties; the cost of obtaining a prescription is too high and if I’d have to see a doctor. |
| Non-User 5 | Bei Verschlechterung der Gesundheit durch die Benutzung des DiGAs (z.B. Magersucht vom EssTagebuch) | If my health were to deteriorate as a result of using the DiGA (e.g., anorexia caused by the food diary). |
| Non-User 6 | Ich mich unwohl dabei fühle intime Gedanken/Anlässe nicht in einem 4 Augengespräch zu teilen | I feel uncomfortable not sharing intimate thoughts or experiences in a one-on-one conversation, |
| Non-User 7 | Sie muss einfach sein. Verständlich, leicht zu benutzen und die Schrift auf dem Endgerät sollte nicht zu klein sein. Nicht "zu viel" Spielerein mit einbauen. Auf garkeinen Fall WERBUNG | It has to be simple. Easy to understand, easy to use, and the text on the device shouldn’t be too small. Don’t include “too many” gimmicks. Under no circumstances should there be ADVERTISEMENTS. |
| Non-User 8 | Die DiGA nicht auf spontane/akute Situationen eingehen kann. Die persönliche Körpersprache kann ein entscheidender Diagnosefaktor sein. | The DiGA can’t address spontaneous or acute situations. A person’s body language can be a crucial diagnostic factor.  I’m still not sure what this is all about. I have nothing against digitization. |
| Non-User 9 | Wenn ich dafür bezahlen muss. | Unless I have to pay for it. |
| Non-User 10 | Nutzung zu kompliziert (technisch) | It’s too complicated to use (technically). |
| Non-User 11 | Info muss durch den Arzt erfolgen, er muss positiv gestimmt seind und das Medium befürworten. | Information must come from the doctor; he must be positive and endorse the platform. |
| Non-User 12 | Größte Faktor ist der Status in der Krankheit. Ab einem gewissen Punkt hat der Patient nicht mehr die Fähigkeit dem ganzen zu folgen und fühlt/wird überfordert, frustriert, selbstzweifel + contraproduktiv | The most important factor is the stage of the illness. At a certain point, the patient is no longer able to keep up with everything and feels overwhelmed, frustrated, and self-doubting—which becomes counterproductive. |
| Non-User 13 | Noch mehr Apps machen den Alltag nicht unbedingt einfacher, sondern erzwigen eher Stress! Stress=Krank | Having even more apps doesn't necessarily make everyday life easier—it actually causes more stress! Stress = Illness |
| Non-User 14 | Kann ich erst beantworten, wenn ich DiGA ausprobiert habe. | I won't be able to answer that until I've tried DiGA. |
| Non-User 15 | Die DiGA muss ohne Google-Server nutzbar sein (Android App) | The DiGA must be usable without Google servers (Android app) |
| Non-User 16 | - unpassende Aufgaben z.B. Hineindenken in unrealistische Situationen; - zu "einfach" | inappropriate tasks, e.g., imagining unrealistic situations; - too “easy” |
| Non-User 17 | - Aufgaben und Inhalte nicht vertieft werden; - Ergebnisse nicht Dokumentiert werden; - Informationen nach Beendigung verloren gehen | Tasks and content are not explored in depth; - Results are not documented; - Information is lost after completion |
